# A novel mathematical model of ATM/p53/NF- *κ*B pathways points to the importance of the DDR switch-off mechanisms

**DOI:** 10.1186/s12918-016-0293-0

**Published:** 2016-08-15

**Authors:** Katarzyna Jonak, Monika Kurpas, Katarzyna Szoltysek, Patryk Janus, Agata Abramowicz, Krzysztof Puszynski

**Affiliations:** 1 Faculty of Automatic Control, Electronics and Computer Science, Silesian University of Technology, Akademicka, Gliwice, 16, 44-100 Poland; 2 Maria Sklodowska-Curie Memorial Cancer Center and Institute of Oncology, Wybrzeze Armii Krajowej, Gliwice, 15, 44-400 Poland

**Keywords:** Mathematical, Model, Deterministic, Stochastic, DNA damage, ATM, Wip1, p53

## Abstract

**Background:**

Ataxia telangiectasia mutated (ATM) is a detector of double-strand breaks (DSBs) and a crucial component of the DNA damage response (DDR) along with p53 and NF- *κ*B transcription factors and Wip1 phosphatase. Despite the recent advances in studying the DDR, the mechanisms of cell fate determination after DNA damage induction is still poorly understood.

**Results:**

To investigate the importance of various DDR elements with particular emphasis on Wip1, we developed a novel mathematical model of ATM/p53/NF- *κ*B pathways. Our results from in silico and in vitro experiments performed on U2-OS cells with Wip1 silenced to 25 % (Wip1-RNAi) revealed a strong dependence of cellular response to DNA damages on this phosphatase. Notably, Wip1-RNAi cells exhibited lower resistance to ionizing radiation (IR) resulting in smaller clonogenicity and higher apoptotic fraction.

**Conclusions:**

In this article, we demonstrated that Wip1 plays a role as a gatekeeper of apoptosis and influences the pro-survival behaviour of cells – the level of Wip1 increases to block the apoptotic decision when DNA repair is successful. Moreover, we were able to verify the dynamics of proteins and transcripts, apoptotic fractions and cells viability obtained from stochastic simulations using in vitro approaches. Taken together, we demonstrated that the model can be successfully used in prediction of cellular behaviour after exposure to IR. Thus, our studies may provide further insights into key elements involved in the underlying mechanisms of the DDR.

**Electronic supplementary material:**

The online version of this article (doi:10.1186/s12918-016-0293-0) contains supplementary material, which is available to authorized users.

## Background

DNA double-strand breaks (DSBs) appear in the non-dividing cells as a result of stress agents activity, what leads to induction of the DNA damage response (DDR) and eventually DNA repair or cell apoptosis [1]. Signal about DSBs is transmitted through ataxia telangiectasia mutated (ATM) – serine/threonine kinase – to p53 cellular tumour antigen and nuclear factor NF- *κ*B. These two transcription factors are responsible for cell fate determination; however, it is still not fully understood how the cell decides about its fate.

Many studies confirmed that DNA lesions and incorrect mechanisms of the DDR may lead to pathological changes transmitted to daughter cells, uncontrolled proliferation and tumour growth [2–7]. It has been reported that an essential role in the DDR is played by protein phosphates, among them Wip1 – a p53-induced protein phosphatase 1D [8]. Wip1 is a crucial component of cancerogenesis and is involved in the ATM/p53 pathways [9–11]. Furthermore, it has been suggested that Wip1 regulates cell-autonomous decline in proliferation of self-renewing cells with advancing age [12].

Investigating the DDR in general and connections between ATM, p53, NF- *κ*B and Wip1 phosphatase in particular is essential for understanding the cellular response to DNA damages. As a result, these studies should help to predict the behaviour of mammalian cells and determine molecules that may become potential drug targets [13]. Although there have been many advances in the field of studying the DDR, the exact mechanism of cell fate determination is still largely unexplored.

To answer the question whether Wip1 plays an important role in cell fate determination and how sensitive the system is to various changes in the ATM/Wip1 modules, we developed a novel mathematical model connecting ATM, Wip1, p53 and NF- *κ*B. A new hybrid model combines stochastic and deterministic approaches and is based on our previously constructed stochastic model of p53/NF- *κ*B pathways [14] and deterministic model of the ATM/p53 pathways [15].

Cell fate is determined by the accumulated levels of p53 and its transcriptional targets, among them Cdk1-inhibitor p21, which initiates the cell cycle arrest [16], and Bax, which triggers the apoptotic events [17]. Overexperession of p21 and Bax has been found in many types of cancers, suggesting their high clinical importance [18–20].

In this article, we describe a specific kinetics of Wip1 after the induction of DSBs by IR. According to in silico and in vitro results, Wip1 plays a role as a gatekeeper in the ATM/p53 system. The phosphatase accumulates in cells upon DSBs induction, in order to “turn off” the DDR system after the successful damage repair. However, if the level of Wip1 stays high, cells may become insensitive to further damages. Hence, the accurate regulation of Wip1 is necessary to repair the system. It has been reported that one of the main regulation factors important in the DDR system involves miRNAs [21, 22]. Therefore, we propose to include miRNA-16 in the model as it plays essential role in restraining proliferation of tumour cells through inhibition of Wip1 transcript [22, 23]. Based on the experimental data, we propose a model containing different regulators of Wip1 transcript, among them miRNA-16 and a stimulator of Wip1 transcription – CREB.

### Model development

To investigate the importance of various components of the DDR system on cell fate determination, we developed a novel mathematical model of the ATM/p53/NF- *κ*B pathways.

Proposed model is based on our previous p53/NF- *κ*B crosstalk model [14] and preliminary ATM pathway model presented in [15]. To cover the experimentally observed Wip1 behaviour, we added regulators of its transcription in form of miRNA-16 and CREB. Our model is one of a few that includes the spatio-temporal regulation of the studied ATM/p53 pathways (we distinguish the nucleus, the cytoplasm and the extracellular matrix) and according to our knowledge the only one that combines that with stochasticity. This extension together with the introduction of p21 and Bax molecules responsible for cell cycle blockade and initiation of apoptosis allow us to simulate a single cell response to DNA damage from the detection system to the mechanisms directly involved in cell fate determination.

**Fig. 1:**
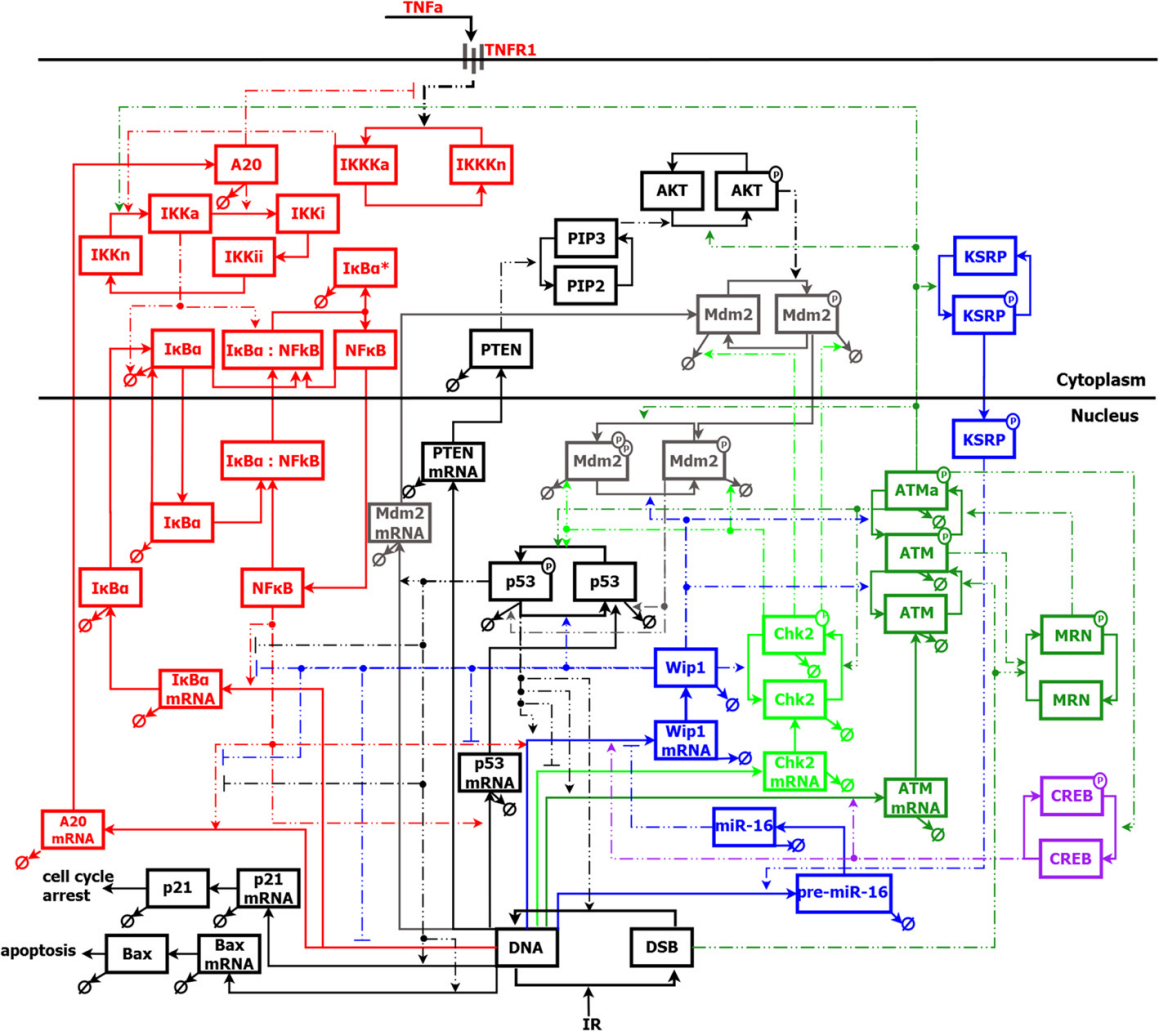
Detailed schematic of the ATM/p53/NF *κ*B pathway model. Active and inactive states of proteins and transcripts (mRNA) are presented. The model distinguishes three compartments: the extracellular matrix, the cytoplasm and the nucleus. There are four main modules of the model: p53 (*black*), NF- *κ*B (*gray*), ATM (*green*) and Wip1 (*blue*). *Dotted lines* with arrow-heads stand for positive regulation between components, *solid lines* for transitions between states of the components, *crossed circles* for degradation of the protein or transcript, and “P” for phosphorylated form of the protein; “a” for ATM stands for fully active form of this kinase

The interactions between molecules are presented in Fig. 1. All of the processes occurring in the system were described with ordinary differential equations (ODEs) disclosed in Additional file 1. Numerical implementation of the model was based on the Haseltine-Rawlings postulate – a hybrid approach used to combine stochastic and deterministic methods [24].

Stochasticity in our model is present as a random gene switching (main source), DNA damage and repair events (as an appearance/disappearance of a particular breakage), and TNF receptors activation/deactivation events.

The model equations were built using basic biochemistry laws: law of mass action and Michaelis-Menten kinetics. Detailed information about the numerical implementation procedure is available in Additional file 2. Detailed information about the model variables and parameters is available in Additional files 3 and 4.

#### Activation of the DNA damage response

In this section, we briefly describe the activation processes of the studied DDR pathways included in the new model. A detailed biological interactions are presented in Additional file 5 and in the references within the articles [14] and [25].

We assume that detection of DSBs is triggered by ATM kinase and Mre11-Rad50-Nbs1 (MRN) complex in the nucleus, as proposed in the previous deterministic model [15]. ATM triggers the activation of p53, Chk2 and indirectly a cytoplasmic form of Mdm2 – a natural inhibitor of p53. Active p53 and Chk2 stimulate the DNA damage signal increasing the activity of proteins involved in ATM and p53 pathways, among them Wip1 phosphatase. Wip1 dephosphorylates main system kinases and transcription factors leading to their inactivation or, as for Mdm2, activation.

We extended our existing p53 model [25] and introduced an additional form of Mdm2 – a multi-phosphorylated inactive nuclear Mdm2. We assumed that two Mdm family members, Mdm2 and MdmX, can be treated as one (Mdm2), what simplify the model description [14, 26].

In the model, we included a complex ATM-dependent regulation of Wip1: through mRNA inhibitor miR-16 (miRNA-16) and its activator KSRP, and Wip1 synthesis activator CREB. Furthermore, Wip1 transcription is up-regulated by p53 and NF- *κ*B. This link between Wip1 and NF- *κ*B leads to the transcriptional inhibition of NF- *κ*B-dependent genes, like these encoding A20, I *κ*B, p53 and Wip1 itself. In contrast, ATM activates NF- *κ*B pathway via the cytoplasmic I *κ* B complex. Finally, the DNA damage signal is transmitted to decision-making proteins: p21 and Bax.

#### Gene switching stochasticity

Stochasticity in the model occurs mainly due to the stochastic gene switching. Genes encoding Wip1 and Chk2 are activated with the probabilities proportional to the amount of p53 transcription factor. Additionally, gene encoding ATM depends on CREB activity. Gene for Wip1 is activated by three factors: p53, CREB and NF- *κ*B. However, Wip1 inhibits its own NF- *κ*B-dependent activation through its negative regulation. Notice that p53 inhibits Chk2 transcription. Deactivation of genes included in the model is assumed as background/spontaneous.

We assumed that each gene has two copies (alleles) with three possible states: both alleles can be active, only one or none of them. In stochastic approach, these states are given by values 2, 1 and 0, respectively, while in deterministic model they are within the range <0,2> that is the mean state for cells population.

#### Cell fate decision

The output of our model is the levels of molecules at a certain time after irradiation and cell fate decision. Following Kracikova et al. findings [27], we implemented a novel p53-dependent threshold mechanism to determine behaviour of cells after introducing DNA damages. For simplification, we considered only two of p53 products: Bax and p21. The lower first threshold determines the cell cycle arrest and is based on the p21 protein level. Due to the fact that p21 is p53-dependent protein and strictly follows its level, the first threshold is also indirectly dependent on active p53.

The higher second threshold is responsible for driving cells to apoptosis and it combines the levels of Bax and active p53. In the model, we used both proteins to reflect the fact that p53 may have also a non-transcriptional influence on the apoptotic decision [28–30].

Briefly, if during the simulation time the levels of active p53 and Bax cross the given thresholds simultaneously, the studied cell is considered as apoptotic. If the p21 level is above the threshold, the cell is considered as one with arrested cycle. If this cell dies before the first division or its cycle is arrested for more than 63.9 h (which prevents large-enough single cell origin colony formation), it is considered as not viable. Detailed description of determining the thresholds is available in Additional file 6.

#### Model fitting and parameters justification

**Fig. 2:**
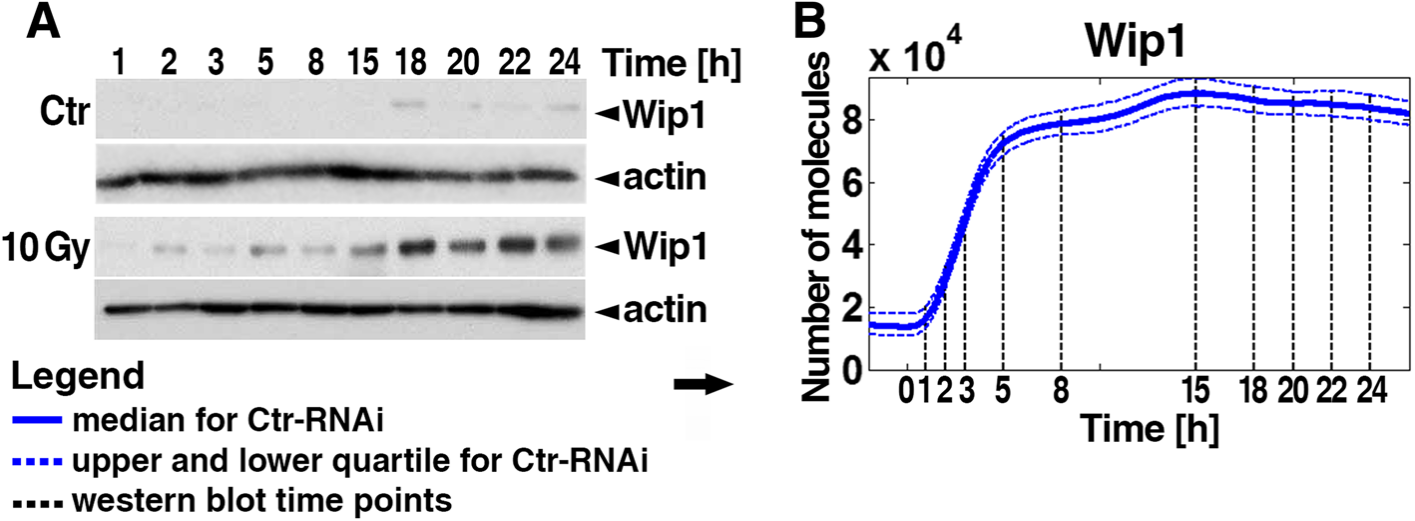
Kinetics of Wip1. **a** Immunoblots of Ctr-RNAi cells show Wip1 kinetic for control cells (“Ctr”) and for cells irradiated with dose of 10 Gy. **b** Results of stochastic simulations on 1000 irradiated cells. *Solid line* stays for median values, *dashed* for 1st and 3rd quartile, while *dashed black lines* for time points when experimental data were gathered

**Fig. 3:**
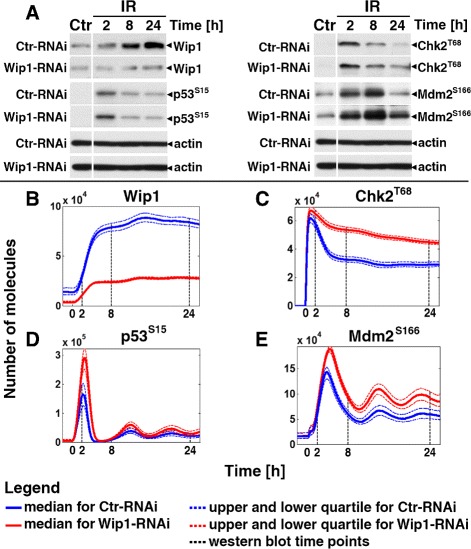
Influence of Wip1 gene silencing on cellular response to DNA damage. **a** Immunoblots of Wip1, active p53, Chk2 and Hdm2 (Mdm2 for mouse) for control and time points 2, 8 and 24 h after exposure to 10 Gy of IR. Experiments were performed for Ctr-RNAi and Wip-RNAi cells. **b**–**e**: Results of stochastic simulations on 1000 cells irradiated with dose of 10 Gy show the levels of various proteins. *Solid line* stays for median values, *dashed* for 1st and 3rd quartile, while *dashed black* lines for time points when experimental data were gathered

**Fig. 4:**
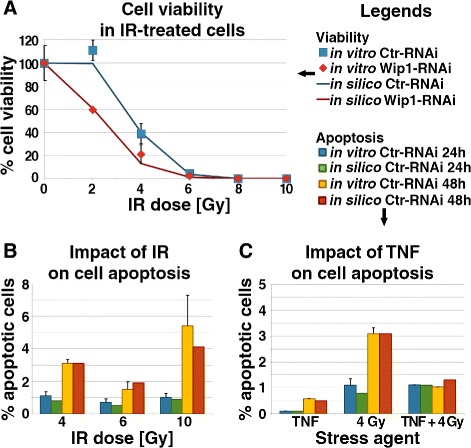
Impact of IR and TNF *α* on cells viability. **a** Percentage of cells viability for Ctr-RNAi and Wip1-RNAi cells after treatment with various doses of IR. **b** Percentage of Ctr-RNAi and Wip1-RNAi apoptotic cells 24 and 48 h after irradiation with various doses of IR. **c** Impact of TNF *α* on fraction size of apoptotic cells. *Note:* Because all *p*-values > 0.05 (for *α*=0.05), most of them > 0.85, there is no reason to reject the null hypothesis that experimental and simulation values are no different

The information about the half-lives of majority of the transcripts and proteins was obtained from the literature or our experiments performed on U2-OS cell line (immunoblots are presented in Additional file 7). The parameters involved in the system activation by IR were obtained by fitting the model to the number of DSBs acquired from our experiments and data from the Kohn and Bohr studies [1]. Transcription and translation rates follow the Levin upper limits [31]. Detailed information about the parameters are available in Additional file 4. The parameters indicated as “fitted” were guessed by a trial-and-error method in such way that they allow the model response to be in agreement with the experimental data presented in Figs. 2, 3, 4b and 4c (training set).

It is known that the parameters estimation is complicated for systems with large number of parameters and mostly for stochastic and hybrid models [32]. One has to remember that complex models with many free parameters suffer from so called “model sloppiness”. It has been demonstrated [33] that even when some individual parameters are poorly constrained (sloppy), collective fitting could result with well-constrained predictions. Moreover, in such case sensitivities spectrum could have the eigenvalues distributed over many decades. In [33], it is postulated that models with constitute rule of sloppy parameters are not exception.

The second common problem of the complex biological network models is a practical non-identifiability – the existence of various parameters sets that have more or less equivalent fitting capability [34]. If the practical non-identifiability exists, the minimum of the performance index in the automatic fitting algorithms is surrounded by a large flat region or multiple local minima of comparable “depth”. Therefore, from the identifiability point of view, time consuming automatic algorithms do not bring any advantage over the trial-and-error method. Even if such an algorithm would be able to find the true minimum, remembering that usually user has to determine the parameters box in which the search take place, the “uniqueness” of such parameter set would be questionable on the ground of biological meaning.

Taking into account this knowledge, we decided to use the trial-and-error method to fit the model to the experimental data. However, we do not claim the uniqueness of our parameters set.

To test the sensitivity of the model to the fitted parameters, we performed sensitivity analysis according to the procedure described in [35] and in Additional file 8. The results show that for the time periods of measurement of apoptotic fractions and viability the model outputs are insensitive to the change of parameters values. This makes our predictions reliable. The results of sensitivity analysis are presented in Additional file 8.

## Results

### Wip1 exhibits a non-oscillatory behaviour after high doses of IR

To investigate the role of Wip1 in cell fate determination, we performed stochastic simulations for 1000 cells and in vitro experiments on U2-OS cells with wild-type expression of Wip1 (Ctr-RNAi) and expression reduced to ca. 25 % of initial value (Wip1-RNAi). Due to the fact that immunoblot experiments were performed on human cancer cell line, we were detecting Mdm2 human analogue – Hdm2.

To investigate the kinetics of Wip1 after exposure to IR, we performed immunoblot assay and stochastic simulations for Ctr-RNAi cells treated with 10 Gy of IR. We compared the levels of Wip1 in irradiated cells to the levels in untreated cells (Fig. 2). Here, our main studies were focused on verification whether Wip1 exhibits an oscillatory response after strong irradiation of cancer cells and if it follows the oscillations (levels) of p53.

In the literature, it has been reported that the transcript of Wip1 follows the levels of p53 and the highest level is observed two hours after irradiation [36, 37]. In the model, we fitted the parameters in a way that the response of Wip1 mRNA is comparable.

We performed immunoblot experiments to verify the kinetics of Wip1 in 24-h time course after irradiation. The data from our biological experiments and simulations are consistent with the reported data about the kinetics of Wip1 in U2-OS cell line [38].

For the control wild-type cells we found the presence of Wip1 protein only 15 h after the initiation of the experiment. The levels of the phosphatase elevated and reached the maximum at 18 h. These clear bands of Wip1 might be an effect of the activation of the DDR in response to DNA damages occurring spontaneously in cells. For the irradiated cells, we observed that the level of Wip1 increased to reach the maximum at around 18 h. These high levels persisted longer than in untreated cells. At 24 h, the levels of Wip1 started to decrease, what suggests that when DSBs are finally repaired by the system Wip1 is being reset in the subsequent cells.

Although some groups reported oscillatory behaviour of Wip1 activated upon DSBs induction [38–40], we observed its non-oscillatory behaviour in U2-OS cells after treatment with 10 Gy of IR during a long 24-h time course. Similar results were obtained by [41] for shorter duration of the experiments. We observed only small rises and falls of Wip1 during the general elevation after IR. Because other research groups reporting oscillatory behaviour of this phosphatase used different damaging agents and/or cell lines, we think that Wip1 response pattern may differ depending on the cell line and damaging agent, maybe even IR doses. This will be the scope of our future research.

It is equivocal if Wip1 phosphatase exhibits oscillatory or non-oscillatory behaviour in general. Here, we decided to use term “rise and fall” of Wip1 levels instead of “oscillations”, which we use rather to describe clear and robust p53/Mdm2 oscillations.

#### Reduction of Wip1 affects the DDR system

To further investigate the influence of IR on Wip1 behaviour, we performed biological experiments on Wip1-RNAi cells treated with 4 Gy, what gave us an answer to the question about the influence of Wip1 on various molecules from ATM/p53/NF- *κ*B pathways. Our simulations and biological experiments (Fig. 3a) clearly demonstrate that the levels of Wip1 decrease around four-fold in Wip1-RNAi cells comparing to Ctr-RNAi.

For p53 (Fig. 3a and d), the highest change between Wip1-RNAi and Ctr-RNAi cells was observed around 2 h after irradiation.

The kinetics of p53 and Mdm2 in Wip1-RNAi did not change drastically comparing to Ctr-RNAi: in both cases these proteins demonstrated high peak of activity after irradiation and then extinguishing oscillations with smaller amplitudes (Fig. 3a, d and e). The response of both proteins in their active forms was stronger in Wip1-RNAi and both stayed longer at these high levels. In contrast, we observed that Chk2 did not oscillate as p53 (Fig. 3a, b and c). The response of Chk2 was stronger in Wip-RNAi than in Ctr-RNAi cells. However, the biggest change was detected after 8 h for irradiated cells, where in Wip1-RNAi cells Chk2 levels were decreasing slower comparing to Ctr-RNAi cells that stabilised at around 18 h. To summarise, we found that silencing Wip1 gene with RNAi enhances the levels of proteins essential in the DDR system.

#### Wip1 maintains cell viability

To analyse cells viability after irradiation and silencing Wip1, we performed biological experiments. Clonogenic abilities (Fig. 4a) of the irradiated cells were measured in vitro and in silico. The clonogenic cell survival assay allows to determinate how many cells are able to proliferate by meaning of at least 30 cells large colony formation during 10 days period. We used received results as training set for stochastic model for Ctr-RNAi and Wip1-RNAi cells. In our study, the inability to proliferate is equivalent to exceeding at least the first threshold described by Kracikova et al. [27]. In the clonogenic cell survival assay, the apoptotic cells and arrested cells are not distinguishable.

Simulated cells were considered as not viable if they died before the end first division or their p21 level was elevated above a given threshold for ∼64 h – these cells were not able to form colonies large enough. After irradiation, reduction of clonogenic potential was observed with increased dose of IR in both Ctr-RNAi and Wip1-RNAi. In cells with silenced Wip1, the clonogenic potential was two-fold lower than in the control for doses of 2 and 4 Gy. After the treatment with doses over 6 Gy, cells lost their clonogenic potential entirely. We observed that doses above 4 Gy successfully stopped cellular division. These data confirmed that Wip1 has a pro-survival effect and regulates apoptosis in mammalian cells, what is consistent with the previous reports [42].

### NF- *κ*B pathway activated before irradiation leads to increased cell survival

One can notice on Fig. 1 that ATM not only activates the p53 pathway but also the NF- *κ*B module through the phosphorylation of IKK. NF- *κ*B itself is also responsible for transcriptional activation of p53. These dependencies increase the number of p53 molecules after irradiation and thus increase the following apoptotic fraction through p53 activation with simultaneous Mdm2 degradation and increase of p53 synthesis. To investigate which of these two interactions is dominant, we performed in vitro and in silico experiments with IR-based activation, only TNF *α*-based NF- *κ*B activation and simultaneous IR- and TNF *α*-based activation.

Here, we focused on a difference in apoptotic fraction in wild-type cells treated with pro-survival agent TNF *α*. Again, we performed stochastic simulations for 1000 cells and biological experiments on U2-OS cell line. For both cases, we irradiated cells 24 h after initiation of the experiment with various doses of IR. For TNF *α*, we used only one dose (10 ng/ml), because testing the effects of various doses of this cytokine was not a subject of our study.

The apoptotic fraction for Ctr-RNAi cells was determined experimentally by flow cytometry assay and then used as a training set for stochastic simulation of 1000 cells per dose (Fig. 4b). Following Kracikova et al. [27], simultaneously elevated levels of p53 and Bax above the given threshold was equivalent to simulated apoptosis of the cells population. In our studies, cells labelled as apoptotic 24 h after irradiation were excluded from the apoptotic fraction at 48 h. Therefore, the total number of cells that died after DSBs induction was a sum of apoptotic cells that did not survive at various time points. We observed that for cells counted 48 h after treatment with IR, the highest apoptotic fraction was observed for 10 Gy, while the lowest was detected for 6 Gy (Fig. 4b). Thus, we concluded that when the irradiation dose is too high causing irreparable damages of DNA chains, Wip1 has no bigger impact on cells viability.

To analyse the impact of NF- *κ*B pathway activation preceding radiation exposure, we performed simulations for Ctr-RNAi cells for three cases: 1) without exposure to IR but with TNF *α* cytokine added in dose of 10 ng/ml for 1 h; 2) without TNF *α* stimulation, but irradiated with 4 Gy; 3) with TNF *α* cytokine stimulation (10 ng/ml for 1 h) and irradiated with dose of 4 Gy two hours after finishing administration of TNF *α*. We counted the apoptotic fraction using flow cytometry technique 24 and 48 h after treatment with IR. We observed (Fig. 4c) small apoptotic fraction for untreated cells with TNF *α* added to the U2-OS cell culture. For irradiated cells with TNF *α* counted 48 h after irradiation, we detected more apoptotic cells but three-fold less than for cells treated with 4 Gy of IR. Our results about the radio-protective effect of TNF *α* added before the irradiation are consistent with the previous reports [43, 44]. TNF administration 6 h after IR increased the number of apoptotic cells in the studied population. Our model predicted 1.3 % of apoptotic cells after 24 h and 4.5 % after 48 h. We investigated dependency between TNF-IR shift and apoptotic fraction in details in [14].

In summary, the results of in silico and in vitro experiments confirmed that the number of apoptotic cells in the population treated with cytokine before irradiation was smaller than in the population exposed only to IR (Fig. 4c). Moreover, we noticed that for TNF *α*-treated cells, they need at least 24 h for the effect of the treatment to be significantly visible.

## Discussion

In this study we constructed a new stochastic model of ATM/p53/NF- *κ*B pathways with Wip1 phosphatase in the main core and additional components responsible for its regulation. Our model is one of a few that includes the spatio-temporal regulation of the studied ATM/p53 pathways [45]. Most of the models of ATM/p53 pathways do not specify the localisation of molecules [39, 46–50]. Contrary, our model includes three compartments contributing to a time delay in the system.

### Wip1 kinetics

In our model, an essential role in cell fate determination after DSBs induction is played by Wip1 protein phosphatase. Similarly, several previously developed models considered the effects of this phosphatase on ATM module alone [47, 50, 51] or on ATM and Mdm2 [52] or Chk2 [48]. In contrast, there are still models that do not include Wip1 as a component of the DDR system [46, 49, 53–56].

Mostly, models that include Wip1 show its oscillatory behavious similarly as for p53s [39, 47, 48, 51, 57]. The only model that does not show oscillatory behaviour of Wip1 is presented in [50]. Biological experiments show different patterns of Wip1 for different cell lines, such as MCF-7 [41], HCT116 [58], RPE [59], BJ fibroblasts [41] and U2-OS [38, 41]. For example, it has been reported that in MCF7 cell line small oscillations of Wip1 are present up to 12 hours after irradiation [39, 51]. Our studies on U2-OS are consistent with studies performed on the same line by other groups [38, 41]. Worth noting is that in our study samples are collected for longer time period: 24 and 48 hours. Other groups often limit the duration of the experiment to 12 hours. We think that it does not fully reflect the kinetics of Wip1. Moreover, we noticed that treatment with different factors, like UV [60], cisplatin [61], 4-hydroxytamoxifen [62] or IR (i.e. in our studies), may have a crucial effect on the kinetics of Wip1 protein. Futhermore, Wip1 regulation may be dose-dependent [40], but we did not perform verification of these observations.

Recent studies [63] indicate that Wip1 activation depends also on a phase of cell cycle. It is another possible reason why sometimes oscillatory behaviour of Wip1 is observed, especially when we take into account the influence of cells synchronisation. These differences of Wip1 behaviour will be a scope of our future work. Using our model we observed dependency which is known from literature – Wip1 is a main deactivation agent for many proteins involved in the DDR pathway. We confirmed that Wip1 is also important in maintaining cells viability. Its accumulation is essential for resumption of cell cycle progression and for deactivation of pro-apoptotic proteins after successful DNA repair [42].

### Influence of complex Wip1 regulation on cell viability

Our previously developed preliminary model of ATM [15] allowed us to predict the overall behaviour of cells treated with IR. However, that model was not fitted to in vitro data or the fitting was not complete. Moreover, observed by us long Wip1 half-lives and elevating levels of mRNA and protein under DSBs induction, resulted in inability of cells to enter apoptosis pathway even if the damages were not repaired. On the other hand, we observed that elimination of Wip1 from the model results in much higher percentage of cells deaths. These cells activated their DNA damage repair processes much later comparing to the wild-type situation. This fact resulted in high difficulties in fitting the model in its previous versions. We found that the solution to this problem may be to introduce miR-16 and a complex Wip1 regulatory mechanisms. miRNAs are found to be important for regulation of the p53 pathway, however there are not many models that include them [64]. Thus, we expanded the preliminary model to obtain results comparable to the biological experiments more accurately [23, 65, 66]. In case of deletion of miR-16 from Wip1 module, we observed increased cells viability (Additional file 9), which is consistent with literature reports [22, 66]. miR-16 is not the only miRNA down-regulating Wip1 [22, 67, 68]. Probably the cumulative activity of more of these molecules cause stronger response of the phophatase.

### Model activation and final response

The DDR system is usually described by the activity of p53 protein and related molecules. Our previous models of p53 pathway [14, 25] and number of studies on dynamics of this module [69–71], describe DNA damages in a simplified way – as a direct input to the model causing activation of p53. In some of the previously described models [51, 55], repair process is also simplified. In [51], p21 was included as a component responsible for cell cycle arrest, allowing DNA repair. Also, in [72] p21-based signalling pathway was added without including details about damage repair. For the apoptosis pathway, Bax was introduced in one of the p53 models [70].

In [73], the authors focused on the order of the first events in the senescence pathway and in [74] on the detailed non-homologous-end-joining (NHEJ) repair. However, we did not intend to built a model of repair and senescence processes. Therefore, similarly as in [51], we included simplified decision making pathways: p21 and Bax. Our assumptions about cell fate are consistent with the experiments from [75], where the higher dosages of IR resulted in increased formation of DSBs and decreased rate if DNA damage repair.

A simplified model described in [39] includes the main feedback loops between Mdm2, Chk2 and Wip1 proteins. In [76], it is suggested that cell fate is not fully dependent on the concentration of active p53 forms, but rather on the dynamics of its oscillations. The results from our experiments and simulations of the stochastic model agree with that suggestion and are true for these cells with wild-type levels of Wip1 and reduced levels to 25 %. The model simulations matched with the dynamics of the main components, like p53, Mdm2 and ATM, obtained from our biological experiments on U2-OS cells. Our model, similarly as in [74], matches the cell-to-cell variability and is able to explain the specific behaviour of the main components of the system.

Some models were developed with simplified ATM/p53 pathways without including their complex regulation [48–50, 56]. In [50], ATM is considered together with a kinase responsible for detection of a single-stranded DNA: ataxia telangiectasia and Rad3-related protein (ATR). In one of the recent models [46], ATM is treated as a parameter that does not change over time. Furthermore, to our knowledge there is not many models including interactions of p53 pathway with other transcription factor responsible for cell fate determination – NF- *κ*B [49, 77].

It has been shown that the pulses occurring in p53 system are dependent on intrinsic DSBs and small oscillations in ATM detector module [39]. In [78], the authors proposed a model with the pulses of p53 maintained by the auto-regulatory positive feedback loop allowing the threshold activation of p53. The activation of the ATM module was shown to exhibit different pattern what possibly depend on the amount of DNA damages per cell [79], similarly as we observed for Wip1.

To date majority of the models on ATM and p53 pathways do not include stochasticity. We have already observed in our previously developed models of p53/NF- *κ*B pathways [14] and ATR/p53 pathways [26] that introducing stochastic effects is crucial for modelling and predicting the behaviour of a single cell and population of cells. The stochasticity for random damage induction and DNA repair was included in the model presented in [80]; however, this model could not reproduce the results concerning the cell-to-cell variability in the pulses of p53 and cell fate. On the other hand, the results of the simulations of stochastic model of NHEJ repair [74] was able to match the observed variability. Our stochastic model has an advantage as the results of simulations on 1000 cells were consistent with the experimental data regarding variability after exposure to IR [76] and the results from our in vitro experiments on U2-OS cell line with the wild-type and reduced levels of Wip1.

### Role of NF- *κ*B pathway in the DDR

Our model shows a cytoprotective effect of TNF *α* on irradiated cells. Number of the apoptotic cells in population treated with this cytokine before irradiation is smaller than in cells irradiated with the same IR dose without TNF *α* treatment. Such influence of TNF, which normally increases apoptotic fractions [81], is observed only in case of treatment with TNF preceding irradiation [43, 44] and was described in our previous work [14].

The results regarding cell fate determination after exposure to IR obtained from the stochastic simulations for different timing of added TNF *α* are in agreement with the previous works. As noted in [77], we observed that the functional consequences of NF- *κ*B activation by addition of TNF *α* probably depend on the number, period and amplitude of the p53 oscillations. The results of simulations and our biological experiments demonstrate that while NF- *κ*B is being activated 3 hours before the induction of DSBs, the levels of proteins responsible for signal detection and transduction decrease comparing to the case where NF- *κ*B pathway was not activated by TNF *α* before irradiation. While analysing in silico results, we saw increased period of p53, Mdm2 and p21 oscillations and decreased amplitude of the pulses. Thus, the treatment resulted in lowered apoptotic fraction.

It has been reported that the effect of the activation of NF- *κ*B pathway by the cytokine may be different depending on the timing of adding TNF *α* [81]. The higher apoptotic fraction was observed for TNF *α* added few hours after treatment with DNA damaging factor. We confirmed it by simulating 1000 cells when TNFa was added six hours after irradiation. When the DDR is already switched on before TNF *α* treatment, the activation of the NF- *κ*B pathway enhances the effect of p53 and results is increased apoptosis of population of cells.

### Limitations of the model

Despite the accuracy of the model predictions, one should remember that our model is based on U2-OS cell line and therefore the results it provides may be inaccurate for other cell lines, DNA damaging agents and/or doses. Especially the number of DSBs after treatment with IR, proteins and mRNAs half-lives, genes activation/inactivation rates and DSBs repair rate may differ between cell lines. Moreover, some cell line-specific modifications, like 25-fold Mdm2 gene amplification in SJSA-1cells or PTEN blockade in MCF-7 or H157 cells, should be included when other cell line is considered. The above mutations in the system can be compensate to some point by changing the model parameters, i.e. degradation rates or total number of active gene alleles. The model does not consider the influence of a phases of cell cycle on cells response to the damage. In our opinion, including the changes in i.e. number of active molecules in various cell cycle phases may be important for cell fate determination.

## Conclusions

The presented stochastic model captures relevant biological aspects of the mechanism of the DDR system based on the ATM/p53/NF- *κ*B network. Stochastic modelling allows to observe differences in a single cell response after treatment with various doses of stress agents and manipulation of the components of the DDR system. These disparities among single cells reacting to the same stimuli under the same conditions are reflected by the apoptotic fractions size and viabilities of the surviving cells.

Thus, the model presented in this study allows investigating the process of determining the cell fate in response to DSBs and provides enhanced explanatory and predictive power. In comparison to our previous works, the current model describes the studied pathways in more details. As we presented in this article, some of the intermediate molecules and interactions omitted in previously developed models provide important delays to the dynamics of the studied system. Moreover, after conducting sensitivity analysis, we noticed that our model is characterised by a relatively large robustness, what allows to use the model for different cell lines and parameters obtained for them. This greatly increases the cognitive and predictive value of our model.

Collectively, we provide clear evidences that the model can be successfully used in the studies on cellular behaviour after introducing DSBs in the system. Thus, we believe that our studies may provide important insights into mechanisms of the complex DDR pathways activated by DSBs induction.

## Methods

**Cell lines** The human osteosarcoma U2-OS cells (ATCC HTB96) were grown in DMEM supplemented with 10 % foetal bovine serum and gentamicin at 37 °C in 5 % CO_2_. For Wip1 down-regulation cells were transduced with a lentivirus containing shRNA sequence specific to PPM1D gene (Wip-RNAi) or a scrambled sequence shRNA (Ctr-RNAi) (sequences from Santa Cruz Biotechnology); transduced cells were selected with puromycin.

**Cell treatments** Treatments started 48 h after cells inoculation; all steps, with exception of irradiation, were performed in a humidified incubator at 37 °C and 5 % CO_2_. Cells were incubated for 60 min in medium containing 10 ng/ml of TNF *α* cytokine (Sigma), irradiated with ionizing radiation or subjected to combined treatment of both cytokine stimulation and irradiation. After cytokine stimulation TNF *α*-containing medium was replaced with fresh TNF *α*-free medium and cells collected at different time points after stimulation; during experiments with combined cytokine and irradiation treatment, after cytokine stimulation TNF *α*-containing medium was replaced with normal one for an additional 3 h before further treatment with radiation. The ionizing radiation (IR) was generated by a linear accelerator (Clinac 600; Varian); cells were exposed to dose range between 2 and 10 Gy (at a dose rate of 1 Gy/min). Irradiated cells were incubated for an additional time (differ during different analyses: 60 min to 24 h for Western blot analyses or 24 and 48 h for cytometry-based analyses) and then collected and analysed. Appropriate controls, either untreated or TNF *α*-treated only, were processed in parallel. To determine the half-life of proteins and transcripts (mRNA), cells were treated with cycloheximide (100 *μ*g/ml) and actinomycin D (5 *μ*g/ml) respectively, and collected at specific time points (15 min to 8 h from the start of treatment).

**Determining the values of the model parameters** The analysis of half-lives of transcripts and proteins was performed using qPCR method and immunoblot, respectively. Degradation rates for the model were counted using the regression analysis with expotential approximation. The formation of DSBs through IR was measured using *γ*-H2AX detection. The detailed information about the procedures of defining the values of the parameters are available in Additional file 10.

**Analysis of cell fate** The analysis of apoptotic fraction (Sub-G1 fraction) was performed with flow cytometry assay. Clonogenic cell survival assay was performed to verify the viability of cells. Details about experimental procedures are available in Additional file 10.

**Computational simulations** Hybrid deterministic-stochastic simulations were performed according to Haseltine and Rawlings [24]. We used Runge-Kutta 4th order algorithm for deterministic and direct Gillespie for stochastic part of the simulations. The simulation time was set to 951000 s, simulation step for 0.1 s, and saving data for every 10 s. We performed 1000 stochastic simulation for each of the experiment. Numerical simulations of the presented model were performed using Solvary (tool developed by Roman Jaksik and Krzysztof Puszynski, Silesian University of Technology).

***χ***^2^**test** Differences between results from biological experiments and simulations were determined by p-values obtained from *χ*^2^ test for two sets of samples. Null hypothesis: experimental and simulation values are no different. Significance level assumed as *α*=0.05.

## Abbreviations

ATM, ataxia telangiectasia mutated; ATR, ataxia telangiectasia mutated and Rad3-related; Cdk1, cyclin-dependent kinase 1; Chk2, checkpoint kinase 2; DDR, DNA damage response; DNA, deoxyribonucleic acid; DSBs, double-strand breaks; Hdm2, human Mdm2 homologue; IR, ionizing radiation; Mdm2, Mouse double minute 2 homolog; MRN, Mre11-Rad50-Nbs1; NF-kB, nuclear factor kappa-light-chain-enhancer of activated B cells; NHEJ, non-homologous end joining; ODE, ordinary differential equation; shRNA, small hairpin RNA; TNFa, tumor necrosis factor alpha; Wip1/PPM1D, protein phosphatase 1D

## Additional files

Additional file 1 Model equations. Transcribed model equations. For the stochastic variables we present reaction propensities, while for deterministic proper ordinary differential equations (ODEs). (PDF 204 kb)

Additional file 2 Numerical implementation. Description and details of numerical implementation. (PDF 132 kb)

Additional file 3 Definition of the variables. Values of the model variables are presented separately for Ctr-RNAi and Wip1-RNAi cells. (PDF 35.2 kb)

Additional file 4 Model parameters. Values and description of model parameters. (PDF 202 kb)

Additional file 5 Biological description. Description of the main biological interactions between the components included in the model. (PDF 96.3 kb)

Additional file 6 Cell fate decision. Description of the procedure of determining the proper thresholds for the decision about the cell fate in the model. (PDF 103 kb)

Additional file 7 Determining the half-life time of proteins included in the model. Immunoblots of total ATM, unphosphorylated p53, p53 phosphorylated on Ser15, Wip1, Hdm2, Hdm2 phosphorylated on Ser166, Chk2. (PNG 738 kb)

Additional file 8 Sensitivity analysis. Description and results of the sensitivity analysis of the fitted parameters. (PDF 1413 kb)

Additional file 9 Impact of miR-16. Impact of miR-16 deletion on irradiated cells. (PNG 2089 kb)

Additional file 10 Methods. Detailed description about the experimental methods described in the article. (PDF 132 kb)
