# Erratum to: A novel mathematical model of ATM/p53/NF-κB pathways points to the importance of the DDR switch-off mechanisms

**DOI:** 10.1186/s12918-016-0340-x

**Published:** 2016-10-21

**Authors:** Katarzyna Jonak, Monika Kurpas, Katarzyna Szoltysek, Patryk Janus, Agata Abramowicz, Krzysztof Puszynski

**Affiliations:** 1 Faculty of Automatic Control, Electronics and Computer Science, Silesian University of Technology, Akademicka 16, 44-100 Gliwice, Poland; 2 Maria Sklodowska-Curie Memorial Cancer Center and Institute of Oncology, Wybrzeze Armii Krajowej 15, 44-400 Gliwice, Poland

After publication of the original article [1], the authors noticed that there is an error with the Funding section. The correct Funding Section should be:

This work was financially supported by the Polish National Science Center (NCN) grants no. DEC-2012/05/D/ST7/02072 (KJ, MK, KP), N N518 287540 (KS, AA) and DEC-2012/05/B/NZ2/01618 (PJ).
