# Supplementary material for: A novel mathematical model of ATM/p53/NF- κB pathways points to the importance of the DDR switch-off mechanisms
Source: BMC Syst Biol. 2016 Aug 15;10:75. doi: 10.1186/s12918-016-0293-0 (PMC4986247; doi:10.1186/s12918-016-0293-0)
Supplement: Additional file 1 — Model equations. Transcribed model equations. For the stochastic variables we present reaction propensities, while for deterministic proper ordinary differential equations (ODEs). (PDF 204 kb) [file 12918_2016_293_MOESM1_ESM.pdf]

A novel mathematical model of ATM/p53/NF-κB pathways points to the importance of the DDR switch-off mechanisms

ADDITIONAL FILE
Model equations

Below we present the model equations distinguishing between the new ones and adopted from our previous model described in [1]. For the stochastic variables we present reaction propensities, while for deterministic proper ordinary differential equations (ODEs). In general, for stochastic reaction the first propensity stays for the increase of the value of proper variable, while the second for the decrease of that value. For details of the numerical implementation of the hybrid stochastic-deterministic algorithm please see the supporting information: Numerical implementation.

1 System activation

1.1 Number of DSBs (DSB):

First propensity describes DSBs creation by radiation of intensity IR, while second one describes DSBs repair induced by $\mathrm{P53}_{pn}$:

$$a_1 = ma_1 IR(t), \quad (1)$$

$$a_2 = mc_1 \frac{DSB(t)}{DSB(t) + mm_1} \frac{pq_2 + pq_3 P53^2_{pn}(t)}{pq_4 + pq_2 + pq_3 P53^2_{pn}(t)}. \quad (2)$$

Number of active receptors ($\boldsymbol{Ra}$): First propensity describes TNFR1 receptors activation by TNFα, while second one their spontaneous deactivation:

$$a_3 = na_7 TNF(t), \quad (3)$$

$$a_4 = nc_3. \quad (4)$$

2 Gene switching

2.1 New propensities

State of ATM gene ($\boldsymbol{G_{atm}}$): First propensity describe the spontaneous, p53-dependent and CREB-dependent gene activation, while second propensity describes spontaneous gene inactivation:

$$a_5 = (mq_1 + pq_3 P53^2_{pn}(t) + mq_2 CREB_{pn}(t)), \quad (5)$$

$$a_6 = mq_3. \quad (6)$$

State of Chk2 gene ($\boldsymbol{G_{chk2}}$): First propensity describes spontaneous gene activation, while second describes spontaneous gene inactivation:

$$a_7 = mq_1, \tag{7}$$
$$a_8 = mq_3. \tag{8}$$

State of Wip1 gene ($\boldsymbol{G_{wip1}}$): First propensity describes spontaneous, p53-dependent, CREB-dependent and NFκB-dependent gene activation with Wip1-dependent blockade of NFκB function, while second one describes spontaneous gene inactivation:

$$\begin{aligned} a_9 &= (pq_2 + pq_3 P53^2_{pn}(t) + mq_2 CREB_{pn}(t) + \\ &+ nq_1 NFKB_n(t) \frac{nm_1}{nm_1 + WIP1_n(t)}), \end{aligned} \tag{9}$$
$$a_{10} = wq_1. \tag{10}$$

State of p53 gene ($\boldsymbol{G_{p53}}$): First propensity describes spontaneous and NFκB-dependent gene activation, while second propensity describes spontaneous gene inactivation:

$$a_{11} = (pq_1 + nq_1 NFKB_n(t) \frac{nm_1}{nm_1 + WIP1_n(t)}), \tag{11}$$
$$a_{12} = pq_5. \tag{12}$$

State of IκBα gene ($\boldsymbol{G_{ikba}}$): First propensity describes NFκB-dependent gene activation, while second one describes IκBα-dependent gene inactivation:

$$a_{13} = nq_1 NFKB_n(t) \frac{nm_1}{nm_1 + WIP1_n(t)}, \tag{13}$$
$$a_{14} = nq_2 IKBA_n(t). \tag{14}$$

State of A20 gene ($\boldsymbol{G_{a20}}$): First propensity describes NFκB-dependent gene activation, while second propensity describes IκBα-dependent gene inactivation:

$$a_{15} = nq_1 NFKB_n(t) \frac{nm_1}{nm_1 + WIP1_n(t)}, \tag{15}$$
$$a_{16} = nq_2 IKBA_n(t). \tag{16}$$

State of Bax gene ($\boldsymbol{G_{bax}}$): First propensity describes spontaneous and p53-dependent gene activation, while second propensity describes spontaneous gene inactivation:

$$a_{17} = (pq_2 + pq_3 P53^2_{pn}(t)), \quad (17)$$
$$a_{18} = pq_4. \quad (18)$$

State of p21 gene (G_{p21}): First propensity describes spontaneous and p53-dependent gene activation, while second propensity describes spontaneous gene inactivation:

$$a_{19} = (pq_2 + pq_3 P53^2_{pn}(t)), \quad (19)$$
$$a_{20} = pq_4. \quad (20)$$

2.2 Propensities adapted from [1]

State of Mdm2 gene (G_{mdm2}): First propensity describes spontaneous and p53-dependent gene activation, while second describes spontaneous gene inactivation:

$$a_{21} = (pq_2 + pq_3 P53^2_{pn}(t)), \quad (21)$$
$$a_{22} = pq_4. \quad (22)$$

State of PTEN gene (G_{pten}): First propensity describes spontaneous and p53-dependent gene activation, while second propensity describes spontaneous gene inactivation:

$$a_{23} = (pq_2 + pq_3 P53^2_{pn}(t)), \quad (23)$$
$$a_{24} = pq_4. \quad (24)$$

3 Translation

3.1 New equations

ATM transcript (ATM_t): First term represents transcription, while second describes mRNA degradation:

$$\frac{d}{dt}ATM_t(t) = ms_1 G_{atm}(t) - md_1 ATM_t(t). \quad (25)$$

Chk2 transcript ($CHK2_t$): First term represents p53-dependent gene transcription, while second describes mRNA degradation:

$$\frac{d}{dt}CHK2_t(t) = ms_2 G_{chk2}(t)\frac{pm_3}{pm_3 + P53_{pn}(t)} - md_3 CHK2_t(t). \quad (26)$$

Wip1 transcript (*$WIP1_t$*): First term represents gene transcription, while second describes miRNA-dependent mRNA degradation. Parameter siR is a switch for silencing of Wip1 with shRNA:

$$\begin{aligned} \frac{d}{dt}WIP1_t(t) &= ws_1G_{wip1}(t) - (wd_1 + siR\ wd_2 + \\ &+ wd_3MiR16(t))WIP1_t(t). \end{aligned} \tag{27}$$

P53 transcript (*$P53_t$*): First term represents gene transcription, while second describes mRNA degradation:

$$\frac{d}{dt}P53_t(t) = ps_1G_{p53}(t) - pd_1P53_t(t). \tag{28}$$

Bax transcript (*BAX_t*): First term represents gene transcription, while second describes mRNA degradation:

$$\frac{d}{dt}BAX_t(t) = bs_1G_{bax}(t) - bd_1BAX_t(t). \tag{29}$$

P21 transcript (*$P21_t$*): First term represents gene transcription, while second describes mRNA degradation:

$$\frac{d}{dt}P21_t(t) = bs_2G_{p21}(t) - bd_3P21_t(t). \tag{30}$$

3.2 Equations adapted from [1]

Mdm2 transcript (*$MDM2_t$*): First term represents gene transcription, while second describes mRNA degradation:

$$\frac{d}{dt}MDM2_t(t) = ps_2G_{mdm2}(t) - pd_6MDM2_t(t). \tag{31}$$

PTEN transcript (*$PTEN_t$*): First term represents gene transcription, while second describes mRNA degradation:

$$\frac{d}{dt}PTEN_t(t) = ps_3G_{pten}(t) - pd_{10}PTEN_t(t). \tag{32}$$

IκBα transcript (*$IKBA_t$*): First term represents p53-dependent gene transcription, while second describes mRNA degradation:

$$\frac{d}{dt}IKBA_t(t) = ns_1 G_{ikba}(t)\frac{pm_3}{pm_3 + P53_{pn}(t)} - nd_2 IKBA_t(t). \tag{33}$$

A20 transcript ($A20_t$): First term represents p53-dependent gene transcription, while second describes mRNA degradation:

$$\frac{d}{dt}A20_t(t) = ns_1 G_{a20}(t)\frac{pm_3}{pm_3 + P53_{pn}(t)} - nd_2 A20_t(t). \tag{34}$$

4 Signal transduction

4.1 New equations

- ATM module

  Nuclear inactive ATM (ATM_n): First terms describe protein synthesis and Wip1-dependent inactivation of phosphorylated form, while next two describe DSB-dependent activation of phosphorylated form and protein degradation:

$$\begin{aligned}\frac{d}{dt}ATM_n(t) &= mt_1 ATM_t(t) + mc_2 WIP1_n(t) ATM_{pn}(t) - \\ &- ATM_n(t) ma_3 \frac{DSB(t)}{DSB(t) + mm_2} - md_2 ATM_n(t). \end{aligned} \tag{35}$$

  Nuclear phosphorylated (intermediate) ATM (ATM_{pn}): First terms describe DSB-dependent activation of intermediate and Wip1-dependent inactivation of fully active form, while next describes MRN-dependent activation to fully active form, Wip1-dependent inactivation of phosphorylated intermediate form and degradation of the protein:

$$\begin{aligned}\frac{d}{dt}ATM_{pn}(t) &= ATM_n(t) ma_3 \frac{DSB(t)}{DSB(t) + mm_2} + \\ &+ mc_2 WIP1_n(t) ATM_{an}(t) - \\ &- ma_4 ATM_{pn}(t) MRN_{pn}(t) - \\ &- mc_2 WIP1_n(t) ATM_{pn}(t) - md_2 ATM_{pn}(t). \end{aligned} \tag{36}$$

  Nuclear fully active ATM (ATM_{an}): First term describes MRN-dependent activation, second Wip1-dependent inactivation and last protein degradation:

$$\begin{aligned}\frac{d}{dt}ATM_{an}(t) &= ma_4 ATM_{pn}(t) MRN_{pn}(t) - \\ &- mc_2 WIP1_n(t) ATM_{an}(t) - \\ &- md_2 ATM_{an}(t). \end{aligned} \tag{37}$$

Nuclear inactive Chk2 (*CHK2$_n$*): First terms describes protein synthesis and Wip1-dependent inactivation of active form, while second describes ATM-dependent activation of inactive form and protein degradation:

$$\begin{aligned}\frac{d}{dt}CHK2_n(t) &= mt_2CHK2_t(t) + mc_3WIP1_n(t)CHK2_{pn}(t) - \\ &- ma_5ATM_{an}(t)CHK2_n(t) - md_4CHK2_n(t). \qquad (38)\end{aligned}$$

Nuclear active Chk2 (*CHK2$_{pn}$*): First term describes ATM-dependent activation, while second Wip1-dependent inactivation and last protein degradation:

$$\begin{aligned}\frac{d}{dt}CHK2_{pn}(t) &= ma_5ATM_{an}(t)CHK2_n(t) - \\ &- mc_3WIP1_n(t)CHK2_{pn}(t) - \\ &- md_4CHK2_{pn}(t). \qquad (39)\end{aligned}$$

Nuclear active MRN complex (*MRN$_{pn}$*): First term describes ATM-dependent and DSBs-dependent activation, while second complex inactivation:

$$\begin{aligned}\frac{d}{dt}MRN_{pn}(t) &= (ma_6ATM_{pn}(t) + ma_7\frac{DSB(t)}{DSB(t) + mm_3} \cdot \\ &\cdot (MRN_{tot} - MRN_{pn}(t)) - mc_4MRN_{pn}(t). \qquad (40)\end{aligned}$$

Nuclear active CREB (*CREB$_{pn}$*): First term describes ATM-dependent activation, while second spontaneous inactivation:

$$\begin{aligned}\frac{d}{dt}CREB_{pn}(t) &= (ma_5ATM_{an}(t))(CREB_{tot} - CREB_{pn}(t)) - \\ &- mc_5CREB_{pn}(t). \qquad (41)\end{aligned}$$

- Wip1 module

  Nuclear Wip1 (*WIP1$_n$*): First term describes protein synthesis, while second its degradation:

$$\frac{d}{dt}WIP1_n(t) = wt_1WIP1_t(t) - wd_4WIP1_n(t). \qquad (42)$$

Cytoplasmic active KSRP (*KSRP$_p$*): First term describes ATM-dependent activation, second export from the nucleus, while third describes inactivation and last import to the nucleus:

$$
\begin{aligned}
\frac{d}{dt}KSRP_p(t) &= wa_1 ATM_{an}(t)\cdot \\
&\cdot\ (KSRP_{tot} - KSRP_p(t) - KSRP_{pn}(t)) + \\
&+\ we_1 KSRP_{pn}(t) - wc_1 KSRP_p(t) - \\
&-\ wi_1 KSRP_p(t). \qquad (43)
\end{aligned}
$$

Nuclear active KSRP (*$KSRP_{pn}$*): First term describes import to the nucleus from the cytoplasm, while second describes export from nucleus:

$$\frac{d}{dt}KSRP_{pn}(t) = wi_1 KSRP_p(t) - we_1 KSRP_{pn}(t). \qquad (44)$$

Prematured miRNA type 16 (*PreMiR16*): First term describes KSRP-dependent synthesis, while second spontaneous degradation:

$$\frac{d}{dt}PreMiR16(t) = ws_2 KSRP_{pn}(t) - wd_5 PreMiR16(t). \qquad (45)$$

Matured miRNA type 16 (*MiR16*): First term describes maturation of miRNA, while second its degradation:

$$\frac{d}{dt}MiR16(t) = wa_2 PreMiR16(t) - wd_6 MiR16(t). \qquad (46)$$

- P53 module

  Cytoplasmic PIP3 (*PIP3*): First term describes spontaneous activation of PIP2, while second PTEN-dependent deactivation:

$$\frac{d}{dt}PIP3(t) = pa_7(PIP_{tot} - PIP3(t)) - pc_3 PTEN(t) PIP3(t). \qquad (47)$$

Bax protein (*BAX*): First term describes protein synthesis, while second its degradation:

$$\frac{d}{dt}BAX(t) = bt_1 BAX_t(t) - bd_2 BAX(t). \qquad (48)$$

p21 protein (*P21*): First term describes protein synthesis, while second its degradation:

$$\frac{d}{dt}P21(t) = bt_2 P21_t(t) - bd_4 P21(t). \qquad (49)$$

4.2 Modified equations from [1] with added interactions with ATM-Wip1 modules

- P53 module

Nuclear inactive p53 ($P53_n$): First terms describe protein synthesis and Wip1-dependent inactivation of active form, while next terms describe spontaneous, ATM-dependent and Chk2-dependent activation, and last two spontaneous and Mdm2-dependent degradation:

$$\begin{aligned}\frac{d}{dt}P53_n(t) &= pt_1P53_t(t) + pc_1P53_{pn}(t)WIP1_n(t) - \\ &- (pa_1 + pa_2\frac{ATM_{an}(t)}{ATM_{an}(t)+pm_1} + \\ &+ pa_3\frac{CHK2_{pn}(t)}{CHK2_{pn}(t)+pm_2})P53_n(t) - \\ &- (pd_2 + pd_3MDM2^2_{pn}(t))P53_n(t). \end{aligned} \tag{50}$$

Nuclear active p53 ($P53_{pn}$): First terms describe spontaneous, ATM-dependent and Chk2-dependent protein activation, while second Wip1-dependent inactivation and next spontaneous and Mdm2-dependent degradation:

$$\begin{aligned}\frac{d}{dt}P53_{pn}(t) &= (pa_1 + pa_2\frac{ATM_{an}(t)}{ATM_{an}(t)+pm_1} + \\ &+ pa_3\frac{CHK2_{pn}(t)}{CHK2_{pn}(t)+pm_2})P53_n(t) - \\ &- pc_1P53_{pn}(t)WIP1_n(t) - \\ &- (pd_4 + pd_5MDM2^2_{pn}(t))P53_{pn}(t). \end{aligned} \tag{51}$$

Cytoplasmic inactive Mdm2 ($MDM2$): First terms describe protein synthesis and inactivation of cytoplasmic phosphorylated form, while next terms describe Akt-dependent activation and spontaneous and Chk2-dependent degradation:

$$\begin{aligned}\frac{d}{dt}MDM2(t) &= pt_2MDM2_t(t) + pc_2MDM2_p(t) - \\ &- pa_4MDM2(t)AKT_p(t) - \\ &- (pd_7 + pd_9\frac{CHK2_{pn}(t)}{CHK2_{pn}(t)+pm_2})MDM2(t). \end{aligned} \tag{52}$$

Cytoplasmic active Mdm2 ($MDM2_p$): First term describes Akt-dependent activation, while second spontaneous inactivation, next stays for protein import to the nucleus and spontaneous and Chk2-dependent degradation:

$$\begin{aligned}\frac{d}{dt}MDM2_p(t) &= pa_4MDM2(t)AKT_p(t) - pc_2MDM2_p(t) - \\ &- pi_1MDM2_p(t) - \\ &- (pd_8 + pd_9\frac{CHK2_{pn}(t)}{CHK2_{pn}(t)+pm_2})MDM2_p(t). \end{aligned} \quad (53)$$

Nuclear active Mdm2 (*MDM2*$_{pn}$): First term describes import of active protein to the nucleus, second Wip1-dependent reactivation of the inactive protein, third describes ATM-dependent inactivation and last spontaneous and Chk2-dependent degradation:

$$\begin{aligned}\frac{d}{dt}MDM2_{pn}(t) &= pi_1MDM2_p(t) + pa_5WIP1_n(t)MDM2_{ppn}(t) - \\ &- pa_6MDM2_{pn}(t)ATM_{an}(t) - \\ &- (pd_8 + pd_9kv\frac{CHK2_{pn}(t)}{CHK2_{pn}(t)+pm_2}) \cdot \\ &\cdot \ MDM2_{pn}(t). \end{aligned} \quad (54)$$

Nuclear inactive Mdm2 (*MDM2*$_{ppn}$): First term describes ATM-dependent inactivation of active form, while second Wip1-dependent reactivation and last spontaneous and Chk2-dependent degradation:

$$\begin{aligned}\frac{d}{dt}MDM2_{ppn}(t) &= pa_6MDM2_{pn}(t)ATM_{an}(t) - \\ &- pa_5WIP1_n(t)MDM2_{ppn}(t) - \\ &- (pd_8 + pd_9kv\frac{CHK2_{pn}(t)}{CHK2_{pn}(t)+pm_2}) \cdot \\ &\cdot \ MDM2_{ppn}(t). \end{aligned} \quad (55)$$

Cytoplasmic active Akt (*AKT*$_p$): First term describes PIP3-dependent and ATM-dependent activation, while second spontaneous inactivation:

$$\begin{aligned}\frac{d}{dt}AKT_p(t) &= pa_8PIP3(t)(1 + pa_9ATM_{an}(t)) \cdot \\ &\cdot \ (AKT_{tot} - AKT_p(t)) - pc_4AKT_p(t). \end{aligned} \quad (56)$$

- NFκB module

  Cytoplasmic IKK (*IKK*:) First term describes formation of IKK from intermediate form of the protein, while second IKKK-dependent and ATM-dependent activation:

$$\frac{d}{dt}IKK(t) = na_4IKK_{ii}(t) -$$

$$- \quad (na_5 IKKK_a(t) + na_6 ATM_{an}(t))IKK(t). \tag{57}$$

Cytoplasmic active IKK ($\boldsymbol{IKK_a}$): First terms describes IKKK-dependent and ATM-dependent activation, while last on A20-dependent inactivation:

$$\begin{aligned} \frac{d}{dt}IKK_a(t) &= (na_5 IKKK_a(t) + na_6 ATM_{an}(t))IKK(t) - \\ &- nc_2 IKK_a(t)\frac{nm_4 + A20(t)}{nm_4}. \end{aligned} \tag{58}$$

4.3 Equations adapted from [1]

- P53 module

  Cytoplasmic PTEN ($\boldsymbol{PTEN}$): First term describes protein synthesis, while second its degradation:

$$\frac{d}{dt}PTEN(t) = pt_3 PTEN_t(t) - pd_{11} PTEN(t). \tag{59}$$

- NFκB module

  Cytoplasmic NFκB ($\boldsymbol{NFKB}$): First two terms describes liberation of NFκB from IκBα and phospho-IκBα with NFκB complexes caused by degradation of the IκBα. Next term stays for IκBα with NFκB complexes formation and the last one for transport of free cytoplasmic NFκB to the nucleus:

$$\begin{aligned} \frac{d}{dt}NFKB(t) &= nk_1 IKBANFKB(t) + nd_1 IKBA_p NFKB(t) - \\ &- nk_2 NFKB(t)IKBA(t) - ni_1 NFKB(t). \end{aligned} \tag{60}$$

Nuclear NFκB ($\boldsymbol{NFKB_n}$): First term describes transport to the nucleus, while second represents depletion of free nuclear NF-κB due to the association with nuclear IκBα:

$$\frac{d}{dt}NFKB_n(t) = ni_1 NFKB(t) - nk_2\ kv\ NFKB_n(t)IKBAn(t). \tag{61}$$

Cytoplasmic IκBα-NFκB complexes ($\boldsymbol{IKBANFKB}$): First two terms describes the formation of the complex and export of the nuclear complexes. Next complexes dissolution due IκBα degradation and the last one describes phosphorylation of IκBα in the complex due to the catalytic activity of IKKa:

$$\begin{aligned}\frac{d}{dt}IKBANFKB(t) &= nk_2NFKB(t)IKBA(t)+\\ &+ ne_2IKBA_nNFKB_n(t)-\\ &- nk_1IKBANFKB(t)-\\ &- na_1IKK_a(t)IKBANFKB(t). \qquad (62)\end{aligned}$$

Cytoplasmic phospho-IκBα with NFκB complexes (*IKBA$_p$NFKB*): First term describes IκBα in complexes phosphorylation by active IKK, while second phosphorylated IκBα degradation and thus liberation of NFκB:

$$\begin{aligned}\frac{d}{dt}IKBA_pNFKB(t) &= na_1IKK_a(t)IKBANFKB(t)-\\ &- nd_1IKBA_pNFKB(t). \qquad (63)\end{aligned}$$

Nuclear IκBα-NFκB complexes (*IKBA$_n$NFKB$_n$*): First term accounts for complex formation, while second describes its transport out of the nucleus:

$$\begin{aligned}\frac{d}{dt}IKBA_nNFKB_n(t) &= nk_2\ kv\ NFKB_n(t)IKBA_n(t)-\\ &- ne_2IKBA_nNFKB_n(t). \qquad (64)\end{aligned}$$

Cytoplasmic IκBα (*IKBA*): First term describes synthesis of the protein, second export from the nucleus, next IKK-dependent activation, formation of the complexes, import to the nucleus and degradation:

$$\begin{aligned}\frac{d}{dt}IKBA(t) &= nt_1IKBA_t(t)+ne_1IKBA_n(t)-\\ &- na_2IKK_a(t)IKBA(t)-nk_2NFKB(t)IKBA(t)-\\ &- ni_2IKBA(t)-nd_3IKBA(t). \qquad (65)\end{aligned}$$

Cytoplasmic phosphorylated IκBα (*IKBA$_p$*): First term stays for IκBα phosphorylation by active IKK, while second for phosphorylated IκBα degradation:

$$\frac{d}{dt}IKBA_p(t) = na_2IKK_a(t)IKBA(t)-nd_1IKBA_p(t). \qquad (66)$$

Nuclear IκBα (*IKBA$_n$*): First term describes import from the cytoplasm, while second complexes formation and last export to the cytoplasm:

$$\begin{aligned}\frac{d}{dt}IKBA_n(t) &= ni_2IKBA(t)-nk_2\ kv\ NFKB_n(t)IKBA_n(t)-\\ &- ne_1IKBA_n(t). \qquad (67)\end{aligned}$$

Cytoplasmic A20 (*A20*): First term describes protein synthesis, while second its degradation:

$$\frac{d}{dt}A20(t) = nt_2A20_t(t) - nd_4A20(t). \tag{68}$$

Cytoplasmic inactive IKKK (*IKKK*): First term describes spontaneous inactivation, while second A20 dependent activation by active receptors:

$$\frac{d}{dt}IKKK(t) = nc_1IKKK_a(t) - na_3Ra(t)IKKK(t)\frac{nm_2}{nm_2 + A20(t)}. \tag{69}$$

Cytoplasmic active IKKK (*IKKK$_a$*): First term describes A20 dependent activation by active receptors and second spontaneous inactivation:

$$\begin{aligned}\frac{d}{dt}IKKK_a(t) &= na_3Ra(t)IKKK(t)\frac{nm_2}{nm_2 + A20(t)} - \\ &- nc_1IKKK_a(t).\end{aligned} \tag{70}$$

Cytoplasmic inactive IKK (*IKK$_i$*): First term describes active IKK inactivation which is A20 dependent and then IKK$_i$ transformation to the intermediate form:

$$\frac{d}{dt}IKK_i(t) = nc_2IKK_a(t)\frac{nm_4 + A20(t)}{nm_4} - na_4IKK_i(t). \tag{71}$$

Cytoplasmic intermediate IKK (*IKK$_{ii}$*): First term describes intermediate IKK formation from IKK$_i$ and second its transformation to the IKK:

$$\frac{d}{dt}IKK_{ii}(t) = na_4IKK_i(t) - na_4IKK_{ii}(t). \tag{72}$$

References

1. Puszynski K, Bertolusso R, Lipniacki T. Crosstalk between p53 and nuclear factor-kappaB systems: pro-and anti-apoptotic functions of NF-kappaB. IET Syst Biol. 2009;3:356–367.
