# Supplementary material for: A novel mathematical model of ATM/p53/NF- κB pathways points to the importance of the DDR switch-off mechanisms
Source: BMC Syst Biol. 2016 Aug 15;10:75. doi: 10.1186/s12918-016-0293-0 (PMC4986247; doi:10.1186/s12918-016-0293-0)
Supplement: Additional file 2 — Numerical implementation. Description and details of numerical implementation. (PDF 132 kb) [file 12918_2016_293_MOESM2_ESM.pdf]

A novel mathematical model of ATM/p53/NF-κB pathways points to the importance of the DDR switch-off mechanisms

ADDITIONAL FILE
Numerical implementation

Our numerical implementation of the hybrid algorithm following Haseltine-Rawlings postulate [1] can be presented in the following steps:

1 For the given time t and given values of all variables (deterministic and stochastic), and knowing both input values, calculate the total propensity function $a^*(t)$ of the occurrence of any possible reaction:

$$\begin{aligned} a^*(t) &= a_1 + a_2 + a_3(M - Ra(t)) + a_4 Ra(t) + \\ &+ a_5(NA_{atm} - G_{atm}(t)) + a_6 G_{atm}(t) + \\ &+ a_7(NA_{chk2} - G_{chk2}(t)) + a_8 G_{chk2}(t) + \\ &+ a_9(NA_{wip1} - G_{wip1}(t)) + a_{10} G_{wip1}(t) + \\ &+ a_{11}(NA_{p53} - G_{p53}(t)) + a_{12} G_{p53}(t) + \\ &+ a_{13}(NA_{ikba} - G_{ikba}(t)) + a_{14} G_{ikba}(t) + \\ &+ a_{15}(NA_{a20} - G_{a20}(t)) + a_{16} G_{a20}(t) + \\ &+ a_{17}(NA_{bax} - G_{bax}(t)) + a_{18} G_{bax}(t) + \\ &+ a_{19}(NA_{p21} - G_{p21}(t)) + a_{20} G_{p21}(t) + \\ &+ a_{21}(NA_{mdm2} - G_{mdm2}(t)) + \\ &+ a_{22} G_{mdm2}(t) + a_{23}(NA_{pten} - G_{pten}(t)) + \\ &+ a_{24} G_{pten}(t). \end{aligned} \quad (1)$$

where NA_i denotes total number of "i-th species", M denotes total number of the cell receptors, Ra denotes the number of active receptors at the time t and G_i "i-th' species" gene state at the time t.

2 Select two random numbers p_1 and p_2 from the uniform distribution on [0, 1].

3 Using the fourth order Runge-Kutta solver evaluate the system of ODEs, accounting for fast reactions, until the time $t + \tau$ such that

$$\ln(p_1) + \int_t^{t+\tau} a^*(s)ds = 0. \quad (2)$$

4 Determine which one of the possible slow reactions occurs at time $t + \tau$ using the inequality

$$\sum_{i=1}^{k-1} a_i(t+\tau) < p_2 a^*(t+\tau) \leq \sum_{i=1}^{k} a_i(t+\tau). \quad (3)$$

where k is the index of the reaction to occur.

5 Replace the time t with $t + \tau$ and if the simulation horizon T is not reached go back to step 1.

To find the time τ from Eq. 1 we regularly sample our ODE system each $\Delta t = 0.1$ sec to determine if the stochastic reaction occurs in the period of time from the last check-time to current one. Of course, in this method some inaccuracies may appear in the stochastic events "firings" the time. Although, because the gene switching as well as receptors activation/inactivation and DSB occurrence/repair times are in the order of minutes, i.e. much larger then the chosen sampling time, Δt inaccuracies should be rare and should not affect the final results.

References

1. Haseltine EL, Rawlings JB. Approximate simulation of coupled fast and slow reactions for stochastic chemical kinetics. J Chem Phys. 2002;117:6959–6969.
