# Supplementary material for: A novel mathematical model of ATM/p53/NF- κB pathways points to the importance of the DDR switch-off mechanisms
Source: BMC Syst Biol. 2016 Aug 15;10:75. doi: 10.1186/s12918-016-0293-0 (PMC4986247; doi:10.1186/s12918-016-0293-0)
Supplement: Additional file 3 — Definition of the variables. Values of the model variables are presented separately for Ctr-RNAi and Wip1-RNAi cells. (PDF 35.2 kb) [file 12918_2016_293_MOESM3_ESM.pdf]

A novel mathematical model of ATM/p53/NF-κB pathways points to the importance of the DDR switch-off mechanisms

ADDITIONAL FILE
Definition of the variables

Generally, in the mathematical model values of the variable are considered as the number of molecules per cell. Variables describing genes indicate the number of active genes with the assumption that there are two alleles of each gene in each cell. DSB and Ra stand for number of DNA double-strand breaks and active receptors, respectively. Values of the variables as presented separately for Ctr-RNAi and Wip1-RNAi cells.

Table 1 Variables for ATM module with DNA double-strand breaks formation.

Variable	Description	Type	Initial value for Ctr-RNAi	Initial value for Wip1-RNAi
DSB	DNA double-strand break	Stochastic	0	0
G_{atm}	State of ATM gene	Stochastic	1	1
ATM_t	ATM transcript	Deterministic	132	132
ATM_n	Inactive nuclear ATM	Deterministic	16130	16130
ATM_{pn}	Phosphorylated nuclear ATM	Deterministic	0	0
ATM_{an}	Fully active nuclear ATM	Deterministic	0	0
G_{chk2}	State of Chk2 gene	Stochastic	1	1
$CHK2_t$	Chk2 transcript	Deterministic	215	215
$CHK2_n$	Inactive nuclear Chk2	Deterministic	71290	71290
$CHK2_{pn}$	Active nuclear Chk2	Deterministic	0	0
MRN_{pn}	Active nuclear MRN complex	Deterministic	0	0
$CREB_{pn}$	Active nuclear CREB	Deterministic	0	0

Table 2 Variables for Wip1 module with miRNA production.

Variable	Description	Type	Initial value for Ctr-RNAi	Initial value for Wip1-RNAi
G_{wip1}	State of Wip1 gene	Stochastic	0	0
$WIP1_t$	Wip1 transcript	Deterministic	29	7
$WIP1_n$	Nuclear Wip1	Deterministic	11770	2918
$KSRP_p$	Active cytoplasmic KSRP	Deterministic	0	0
$KSRP_{pn}$	Active nuclear KSRP	Deterministic	0	0
PreMiR16	pre-miRNA type 16	Deterministic	0	0
MiR16	miRNA type 16	Deterministic	0	0

Table 3 Variables for determination of cell fate

Variable	Description	Type	Initial value for Ctr-RNAi	Initial value for Wip1-RNAi
G_{bax}	State of Bax gene	Stochastic	0	0
BAX_t	Bax transcript	Deterministic	27	27
BAX	Bax protein	Deterministic	7691	7683
G_{p21}	State of p21 gene	Stochastic	0	0
$P21_t$	p21 transcript	Deterministic	16	16
P21	p21 protein	Deterministic	7993	7990

Table 4 Variables for p53 module with positive PTEN feedback and negative Mdm2 feedback.

Variable	Description	Type	Initial value for Ctr-RNAi	Initial value for Wip1-RNAi
G_{p53}	State of p53 gene	Stochastic	1	1
$P53_t$	p53 transcript	Deterministic	353	353
$P53_n$	Inactive nuclear p53	Deterministic	30615	30478
$P53_{pn}$	Active nuclear p53	Deterministic	6097	6251
G_{mdm2}	State of Mdm2 gene	Stochastic	0	0
$MDM2_t$	Mdm2 transcript	Deterministic	31	31
MDM2	Inactive cytoplasmic Mdm2	Deterministic	34496	34596
$MDM2_p$	Active cytoplasmic Mdm2	Deterministic	15485	15511
$MDM2_{pn}$	Active nuclear Mdm2	Deterministic	163854	164176
$MDM2_{ppn}$	Inactive nuclear Mdm2	Deterministic	0	0
G_{pten}	State of PTEN gene	Stochastic	0	0
$PTEN_t$	PTEN transcript	Deterministic	16	16
PTEN	Cytoplasmic PTEN	Deterministic	31225	31261
PIP3	Cytoplasmic active PIP3	Deterministic	189556	189389
AKT_p	Cytoplasmic active Akt	Deterministic	1378	1376

Table 5 Variables for NFκB module.

Variable	Description	Type	Initial value for Ctr-RNAi	Initial value for Wip1-RNAi
Ra	Receptors activation	Stochastic	0	0
NFKB	Cytoplasmic NFκB	Deterministic	174	174
$NFKB_n$	Nuclear NFκB	Deterministic	184	177
IKBANFKB	Cytoplasmic IκBα-NFκB complex	Deterministic	99629	99599
$IKBA_pNFKB$	Cyt. active IκBα-NFκB complex	Deterministic	0	0
$IKBA_nNFKB_n$	Nuclear IκBα-NFκB complex	Deterministic	35	35
G_{ikba}	State of IκBα gene	Stochastic	0	0
$IKBA_t$	IκBα transcript	Deterministic	35	36
IKBA	Cytoplasmic IκBα	Deterministic	819	840
$IKBA_p$	Cytoplasmic active IκBα	Deterministic	0	0
$IKBA_n$	Nuclear IκBα	Deterministic	1077	1115
G_{a20}	State of A20 gene	Stochastic	0	0
$A20_t$	A20 transcript	Deterministic	35	36
A20	Cytoplasmic A20	Deterministic	14659	14775
IKKK	Cytoplasmic IKKK	Deterministic	10000	10000
$IKKK_a$	Cytoplasmic active IKKK	Deterministic	0	0
IKK	Cytoplasmic IKK	Deterministic	200000	200000
IKK_a	Cytoplasmic active IKK	Deterministic	0	0
IKK_i	Cytoplasmic inactive IKK	Deterministic	0	0
IKK_{ii}	Cyt. intermediate form of IKK	Deterministic	0	0
