# Supplementary material for: A novel mathematical model of ATM/p53/NF- κB pathways points to the importance of the DDR switch-off mechanisms
Source: BMC Syst Biol. 2016 Aug 15;10:75. doi: 10.1186/s12918-016-0293-0 (PMC4986247; doi:10.1186/s12918-016-0293-0)
Supplement: Additional file 4 — Model parameters. Values and description of model parameters. (PDF 202 kb) [file 12918_2016_293_MOESM4_ESM.pdf]

A novel mathematical model of ATM/p53/NF-κB pathways points to the importance of the DDR switch-off mechanisms

ADDITIONAL FILE
Model parameters

Degradation rates of some transcripts (for ATM, Chk2, Wip1, p53, Mdm2) and proteins (ATM, Chk2, p53, Mdm2) were acquired from half-life times from immunoblot experiments performed on U2-OS cell line. The results are in agreement with Sharova *et al.* [1] and majority of remaining degradation rates was taken from this work. Bax and p21 proteins half-life times were reported to reach around 5.5 hours [2] and 80 min [3], respectively. Half-life time for PTEN was stated to be equal to 10 hours [4], for A20 8 hours [5] and IκBα bounded to NF-κB over 9.5 hours [6]. Interestingly, free IκBα is degraded very fast comparing to other proteins: its half-life time is equal to 10 min [6]. The parameters involved in the system activation by IR were obtained by fitting the model to the number of DSBs acquired from our experiments, indicating 24 DSBs for irradiation with 4 Gy, and from experiments performed by Kohn and Bohr reporting 19 DSBs for 2 Gy and 29 for 8 Gy [7]. The parameters involved in DSB repair were calculated by fitting the model to DSB repair curve in U2-OS cells reported by Malewicz *et al.* [8]. For TNFα based activation of NF-κB module, we used receptor activation/inactivation rates reported by Grell *et al.* [9]: receptor activation rate about 2/min at 10 ng dose; receptor inactivation rate: dissociation $t_{1/2}$=33 min, internalization $t_{1/2}$=10-20 min. Transcription and translation rates follow Levin upper limits [10]. When the gene is active, transcription proceeds with a constant rate and speed of mRNA polymerase – around 40 nt/sec. Characteristic minimum spacing between the neighbouring polymerases is 250 nt, what is assumed as an upper limit for transcription rate of a single allele equal to 40/250 = 0.16 mRNA/s [10]. Similarly, knowing the maximum translation rate and the minimum space between the ribosomes, we obtain the upper limit for translation efficiency equal to 0.5 protein/mRNA/sec. The genes activation/inactivation and transcription/translation coefficients follow Bengtsson *et al.* results [11]. It is reported that if the gene is active, the number of mRNA molecules in the single cell can reach $3 \cdot 10^4$. These numbers were chosen in such a manner that the total number of p53 and Mdm2 proteins at the steady state without any input signals follow the results of Wang *et al.* [12] for U2-OS cells, i.e. $35 - 39 \cdot 10^3$ p53 molecules and $200 - 250 \cdot 10^3$ Mdm2 molecules. The total numbers of PIP and Akt molecules per cell were obtained from Gray *et al.* [13] and Atrih *et al.* [14], respectively.

For all the model parameters n stands for number of molecules or entities such as active genes, and *MM* stands for Michaelis-Menten constant. Origin of the values of the parameters is indicated in the column *Details*: *experiments* indicates that the certain value is derived from biological experiments performed by the authors;

assumed indicates the authors assumption; *fitted* stays for the value received from the model fitting to the biological data acquired by the authors. When the parameter is taken from the literature or fitted to the literature data, the proper reference is provided.

Table 1 Parameters of the model equations for activation of the model and ATM module.

Parameter	Description	Value	Details
NA_{atm}	Number of ATM alleles	$2\ n$	assumed
NA_{chk2}	Number of Chk2 alleles	$2\ n$	assumed
$CREB_{tot}$	Total number of CREB molecules	$100000\ n$	assumed
MRN_{tot}	Total number of MRN molecules	$10000\ n$	assumed
ma_1	DSB damage caused by IR	$0.58\ \frac{n \cdot min}{s \cdot Gy}$	fitted [7]
ma_3	ATM activation by DSB formation	$1.5 \cdot 10^{-3}\ s^{-1}$	fitted
ma_4	ATM fully activation by MRN complex formation	$5 \cdot 10^{-6}\ n^{-1}s^{-1}$	fitted
ma_5	Chk2 and CREB activation by ATM	$1 \cdot 10^{-7}\ n^{-1}s^{-1}$	fitted
ma_6	MRN complex activation by ATM	$4 \cdot 10^{-7}\ n^{-1}s^{-1}$	fitted
ma_7	MRN complex activation by DSB	$5 \cdot 10^{-5}\ s^{-1}$	fitted
mc_1	DSB repair rate	$3.8 \cdot 10^{-3}\ ns^{-1}$	fitted [8]
mc_2	ATM inactivation by Wip1	$6 \cdot 10^{-8}\ n^{-1}s^{-1}$	fitted
mc_3	Chk2 inactivation by Wip1	$1 \cdot 10^{-8}\ n^{-1}s^{-1}$	fitted
mc_4	MRN complex inactivation	$2 \cdot 10^{-3}\ s^{-1}$	fitted
mc_5	CREB complex inactivation	$1 \cdot 10^{-3}\ s^{-1}$	fitted
md_1	ATM transcript degradation	$3.77 \cdot 10^{-5}\ s^{-1}$	experiments
md_2	ATM protein degradation	$4.11 \cdot 10^{-5}\ s^{-1}$	experiments
md_3	Chk2 transcript degradation	$4.18 \cdot 10^{-5}\ s^{-1}$	experiments
md_4	Chk2 protein degradation	$3.02 \cdot 10^{-5}\ s^{-1}$	experiments
mm_1	MM for DSB repair	$10\ n$	fitted
mm_2	MM for ATM activation by DSB	$1\ n$	fitted
mm_3	MM for MRN complex activation by DSB	$1\ n$	fitted
mq_1	Chk2 gene activation	$3 \cdot 10^{-3}\ s^{-1}$	fitted
mq_2	Wip1 gene activation by CREB	$3 \cdot 10^{-8}\ n^{-1}s^{-1}$	fitted
mq_3	Chk2 gene inactivation	$3 \cdot 10^{-3}\ s^{-1}$	fitted
ms_1	ATM synthesis	$5 \cdot 10^{-3}\ s^{-1}$	fitted [10]
ms_2	Chk2 synthesis	$0.01\ s^{-1}$	fitted [10]
mt_1	ATM transcription	$5 \cdot 10^{-3}\ s^{-1}$	fitted [10]
mt_2	Chk2 transcription	$0.01\ s^{-1}$	fitted [10]

Table 2 Parameters of the model equations for Wip1 module.

Parameter	Description	Value	Details
siR	wip-sh switch off/on	0 or 1	switch
NA_{wip1}	Number of Wip1 alleles	$2\ n$	assumed
$KSRP_{tot}$	Total number of KSRP molecules	$100000\ n$	assumed
wa_1	KSRP activation by ATM	$2 \cdot 10^{-9}\ n^{-1}s^{-1}$	fitted
wa_2	miR-16 maturation	$1 \cdot 10^{-4}\ s^{-1}$	fitted
wc_1	KSRP deactivation	$5 \cdot 10^{-5}\ s^{-1}$	fitted
wd_1	Wip1 mRNA spontaneous degradation	$2.8 \cdot 10^{-4}\ s^{-1}$	experiments
wd_2	Wip1 mRNA degradation caused by siRNA	$8.656 \cdot 10^{-4}\ s^{-1}$	fitted
wd_3	Wip1 mRNA degradation caused by miR-16	$8.67 \cdot 10^{-7}\ n^{-1}s^{-1}$	fitted
wd_4	Wip1 protein degradation rate	$3.96 \cdot 10^{-5}\ s^{-1}$	experiments
wd_5	pre-miR degradation rate	$2 \cdot 10^{-5}\ s^{-1}$	fitted
wd_6	miR-16 degradation	$5 \cdot 10^{-5}\ s^{-1}$	fitted
we_1	KSRP nuclear export	$5 \cdot 10^{-5}\ s^{-1}$	fitted
wi_1	KSRP nuclear import	$5 \cdot 10^{-5}\ s^{-1}$	fitted
wq_1	Wip1 gene deactivation	$3 \cdot 10^{-3}\ s^{-1}$	fitted
ws_1	WIP1 synthesis rate	$8.652 \cdot 10^{-2}\ s^{-1}$	fitted [10]
ws_2	pre-miR-16 production rate	$1 \cdot 10^{-7}\ s^{-1}$	fitted
wt_1	WIP1 translation rate	$1.6 \cdot 10^{-2}\ s^{-1}$	fitted [10]

Table 3 Parameters of the model equations for cell fate.

Parameter	Description	Value	Details
NA_{bax}	Number of Bax alleles	$2\ n$	assumed
NA_{p21}	Number of p21 alleles	$2\ n$	assumed
bd_1	Bax mRNA degradation	$2.87 \cdot 10^{-5}\ s^{-1}$	[1]
bd_2	Bax degradation	$3.5 \cdot 10^{-5}\ s^{-1}$	[2]
bs_1	Bax mRNA synthesis	$0.01\ s^{-1}$	fitted [10]
bt_1	Bax translation	$0.01\ s^{-1}$	fitted [10]
bd_3	p21 mRNA degradation	$9.5 \cdot 10^{-5}\ s^{-1}$	[1]
bd_4	p21 degradation	$1.44 \cdot 10^{-4}\ s^{-1}$	[3]
bs_2	p21 mRNA synthesis	$0.02\ s^{-1}$	fitted [10]
bt_2	p21 translation	$0.07\ s^{-1}$	fitted [10]

Table 4 Parameters of the model equations for p53 module.

Parameter	Description	Value	Details
NA_{mdm2}	Number of Mdm2 alleles	$2\ n$	assumed
NA_{p53}	Number of p53 alleles	$2\ n$	assumed
NA_{pten}	Number of PTEN alleles	$2\ n$	assumed
AKT_{tot}	Total amount of Akt molecules	$34000\ n$	[14]
PIP_{tot}	Total amount of PIP molecules	$800000\ n$	[13]
kv	Cytoplasmic to nuclear volume ratio	5	[15]
pa_1	p53 spontaneous activation	$8.6 \cdot 10^{-5}\ s^{-1}$	fitted
pa_2	p53 activation by ATM	$5 \cdot 10^{-5}\ s^{-1}$	fitted
pa_3	p53 activation by Chk2	$5 \cdot 10^{-5}\ s^{-1}$	fitted
pa_4	Mdm2 phosphorylation by AKT	$3 \cdot 10^{-7}\ n^{-1}s^{-1}$	fitted
pa_5	Nuclear Mdm2 reactivation by Wip1	$1.2 \cdot 10^{-7}\ n^{-1}s^{-1}$	fitted
pa_6	Nuclear Mdm2 inactivation by ATM	$2 \cdot 10^{-7}\ n^{-1}s^{-1}$	fitted
pa_7	PIP3 activation	$5 \cdot 10^{-5}\ s^{-1}$	[15]
pa_8	AKT activation by PIP	$4 \cdot 10^{-11}\ n^{-1}s^{-1}$	fitted
pa_9	AKT activation by ATM	$7 \cdot 10^{-4}\ n^{-1}$	fitted
pc_1	p53 inactivation by Wip1	$1.6 \cdot 10^{-9}\ n^{-1}s^{-1}$	fitted
pc_2	Mdm2 dephosphorylation rate	$1 \cdot 10^{-4}\ s^{-1}$	[15]
pc_3	PIP inactivation by PTEN	$5.17 \cdot 10^{-9}\ n^{-1}s^{-1}$	fitted
pc_4	AKT inactivation	$1.8 \cdot 10^{-4}\ s^{-1}$	fitted
pd_1	p53 transcript degradation	$8.3 \cdot 10^{-5}\ s^{-1}$	experiments
pd_2	p53 protein spontaneous degradation	$5.97 \cdot 10^{-5}\ s^{-1}$	experiments
pd_3	p53 degradation by Mdm2	$1.45 \cdot 10^{-13}\ n^{-2}s^{-1}$	fitted
pd_4	p53p protein spontaneous degradation	$2.41 \cdot 10^{-5}\ s^{-1}$	experiments
pd_5	p53p degradation by Mdm2	$1.45 \cdot 10^{-14}\ n^{-2}s^{-1}$	fitted
pd_6	Mdm2 transcript degradation	$7.93 \cdot 10^{-5}\ s^{-1}$	experiments
pd_7	Mdm2 protein spontaneous degradation	$4.79 \cdot 10^{-5}\ s^{-1}$	experiments
pd_8	Mdm2p protein spontaneous degradation	$7.04 \cdot 10^{-5}\ s^{-1}$	experiments
pd_9	Mdm2 degradation by Chk2	$4.5 \cdot 10^{-5}\ s^{-1}$	fitted
pd_{10}	PTEN transcript degradation rate	$3 \cdot 10^{-4}\ s^{-1}$	[1]
pd_{11}	PTEN degradation rate	$5 \cdot 10^{-5}\ s^{-1}$	[4]
pi_1	Mdm2 nuclear import	$7.5 \cdot 10^{-4}\ s^{-1}$	fitted
pm_1	MM for p53 activation by ATM	$1\ n$	fitted
pm_2	MM for Chk2 influence on the system	$1\ n$	fitted
pm_3	MM for p53 caused translation inhibition	$55400\ n$	fitted
pq_1	p53 gene spontaneous activation	$2.1 \cdot 10^{-3}\ s^{-1}$	fitted
pq_2	Spont. activation of Mdm2 and PTEN genes	$1 \cdot 10^{-4}\ s^{-1}$	[15]
pq_3	p53-dep. activation of Mdm2 and PTEN genes	$5.87 \cdot 10^{-13}\ n^{-2}s^{-1}$	fitted
pq_4	Mdm2 and PTEN genes inactivation	$3 \cdot 10^{-3}\ s^{-1}$	[15]
pq_5	p53 gene spontaneous inactivation	$2.1 \cdot 10^{-3}\ s^{-1}$	fitted
ps_1	Synthesis of p53 mRNA	$2.9 \cdot 10^{-2}\ s^{-1}$	fitted [10]
ps_2	Synthesis of Mdm2 mRNA	$3.1 \cdot 10^{-2}\ s^{-1}$	fitted [10]
ps_3	Synthesis of PTEN mRNA	$0.06\ s^{-1}$	[15, 10]
pt_1	p53 translation rate	$0.35\ s^{-1}$	fitted [10]
pt_2	Mdm2 translation rate	$0.47\ s^{-1}$	[15, 10]
pt_3	PTEN translation rate	$0.1\ s^{-1}$	[15, 10]

Table 5 Parameters of the model equations for NF-κB module.

Parameter	Description	Value	Details
NA_{a20}	Number of A20 alleles	$2\ n$	assumed
NA_{ikba}	Number of IκBα alleles	$2\ n$	assumed
M	Total number of receptors	$1000\ n$	[15]
na_1	IKKa mediated IκBα in complexes phosph.	$5 \cdot 10^{-7}\ n^{-1}s^{-1}$	[15]
na_2	IKKa mediated IκBα phosphorylation	$1 \cdot 10^{-7}\ n^{-1}s^{-1}$	[15]
na_3	IKKK activation	$1 \cdot 10^{-4}\ n^{-1}s^{-1}$	[15]
na_4	IKKii transformation	$5 \cdot 10^{-4}\ s^{-1}$	[15]
na_5	IKK activation	$5 \cdot 10^{-6}\ n^{-1}s^{-1}$	[15]
na_6	Activation of IKK by ATM	$5 \cdot 10^{-10}\ n^{-1}s^{-1}$	fitted
na_7	TNF caused receptor activation	$4 \cdot 10^{-6}\ \frac{ml}{ng \cdot s}$	[9]
nc_1	IKKK inactivation rate	$0.01\ s^{-1}$	[15]
nc_2	IKK inactivation rate	$3 \cdot 10^{-3}\ s^{-1}$	[15]
nc_3	Receptor inactivation rate	$6 \cdot 10^{-4}\ s^{-1}$	[9]
nd_1	Phosphorylated IκBα degradation	$2.31 \cdot 10^{-3}\ s^{-1}$	fitted
nd_2	A20 and IκBα transcript degradation	$1.28 \cdot 10^{-4}\ s^{-1}$	[1]
nd_3	Free IκBα degradation	$1.16 \cdot 10^{-3}\ s^{-1}$	[6]
nd_4	A20 degradation rate	$2.4 \cdot 10^{-5}\ s^{-1}$	[5]
ne_1	IκBα nuclear export	$5 \cdot 10^{-3}\ s^{-1}$	[15]
ne_2	IκBα-NFκB nuclear export	$0.05\ s^{-1}$	[15]
ni_1	NFκB nuclear import	$0.01\ s^{-1}$	[15]
ni_2	IκBα nuclear import	$8.7 \cdot 10^{-3}\ s^{-1}$	fitted
nk_1	IκBα in complex with NFκB degradation	$2 \cdot 10^{-5}\ s^{-1}$	[6]
nk_2	IκBα-NFκB association rate	$1.76 \cdot 10^{-6}\ n^{-1}\ s^{-1}$	fitted
nm_1	MM for Wip1 influence on NFκB dep. genes	$100000\ n$	fitted
nm_2	MM for IKKK inhibition by A20	$10000\ n$	[15]
nm_4	MM for IKKa inactivation by A20	$10000\ n$	[15]
nq_1	NFκB dependent genes activation	$1.68 \cdot 10^{-7}\ n^{-1}\ s^{-1}$	fitted
nq_2	IκBα and A20 genes inactivation	$1 \cdot 10^{-6}\ n^{-1}\ s^{-1}$	fitted
ns_1	A20 and IκBα mRNA synthesis	$0.1\ s^{-1}$	[15, 10]
nt_1	IκBα translation	$0.0836\ s^{-1}$	fitted [10]
nt_2	A20 translation	$0.01\ s^{-1}$	fitted [10]

References

1. Sharova LV, Sharov AA, Nedorezov T, Piao Y, Shaik N, Ko MS. Database for mRNA Half-Life of 19 977 Genes Obtained by DNA Microarray Analysis of Pluripotent and Differentiating Mouse Embryonic Stem Cells. DNA Res. 2009;16:45–58.
2. Magal SS, Jackman A, Ish-Shalom S, Botzer LE, Gonen P, Schlegel R, et al. Downregulation of Bax mRNA expression and protein stability by the E6 protein of human papillomavirus 16. J Gen Virol. 2005;86:611–621.
3. Lee H, Zeng SX, Lu H. UV Induces p21 rapid turnover independently of ubiquitin and Skp2. J Biol Chem. 2006;281:26876–26883.
4. Yang Y, Zhou F, Fang Z, Wang L, Li Z, Sun L, et al. Post-transcriptional and post-translational regulation of PTEN by transforming growth factor-beta1. J Cell Biochem. 2009;106:1102–1112.
5. Werner SL, Kearns JD, Zadorozhnaya V, Lynch C, O'Dea E, Boldin MP, et al. Encoding NF-kB temporal control in response to TNF: distinct roles for the negative regulators IkBa and A20. Genes Dev. 2008;22:2093–2101.
6. O'Dea EL, Barken D, Peralta RQ, Tran KT, Werner SL, Kearns JD, et al. A homeostatic model of IkB metabolism to control constitutive NF-kB activity. Mol Syst Biol. 2007;3:1–7.
7. Kohn KW, Bohr VA. Genomic Instability and DNA Repair. In: The Cancer Handbook. vol. 1. London: Nature Publishing Group, Macmillan Publishing; 2002. p. 87–106.
8. Malewicz M, Kadkhodaei B, Kee N, Volakakis N, Hellman U, Viktorsson K, et al. Essential role for DNA-PK-mediated phosphorylation of NR4A nuclear orphan receptors in DNA double-strand break repair. Genes Dev. 2011;25:2031–2040.
9. Grell M, Wajant H, Zimmermann G, Scheurich P. The type 1 receptor (CD120a) is the high-affinity receptor for soluble tumor necrosis factor. Proc Natl Acad Sci U S A. 1998;95:570–575.
10. Levin B. Genes. vol. VII. Oxford: Oxford University Press; 2000.
11. Bengtsson M, Hemberg M, Rorsman P, Stahlberg A. Quantification of mRNA in single cells and modelling of RT-qPCR induced noise. BMC Mol Biol. 2008;9(63).
12. Wang YV, Wade M, Wong E, Li YC, Rodewald LW, Wahl GM. Quantitative analyses reveal the importance of regulated Hdmx degradation for p53 activation. Proc Natl Acad Sci U S A. 2007;104:12365–12370.
13. Gray A, Olsson H, Batty IH, Priganica L, Downes CP. Nonradioactive methods for the assay of phosphoinositide 3-kinases and phosphoinositide phosphatases and selective detection of signaling lipids in cell and tissue extracts. Anal Biochem. 2003;313:234–245.
14. Atrih A, Turnock D, Sellar G, Thompson A, Feuerstein G, Ferguson, MA, et al. Stoichiometric quantification of Akt phosphorylation using LC-MS/MS. J Proteome Res. 2009;9:737–751.
15. Puszynski K, Bertolusso R, Lipniacki T. Crosstalk between p53 and nuclear factor-kappaB systems: pro-and anti-apoptotic functions of NF-kappaB. IET Syst Biol. 2009;3:356–367.
