# Supplementary material for: A novel mathematical model of ATM/p53/NF- κB pathways points to the importance of the DDR switch-off mechanisms
Source: BMC Syst Biol. 2016 Aug 15;10:75. doi: 10.1186/s12918-016-0293-0 (PMC4986247; doi:10.1186/s12918-016-0293-0)
Supplement: Additional file 5 — Biological description. Description of the main biological interactions between the components included in the model. (PDF 96.3 kb) [file 12918_2016_293_MOESM5_ESM.pdf]

A novel mathematical model of ATM/p53/NF-κB pathways points to the importance of the DDR switch-off mechanisms

ADDITIONAL FILE
Biological description

After DSBs detection ATM is auto-phosphorylated on Ser1981 and dissociates from inactive homodimer to active monomers [1]. Further auto-phosphorylation of ATM on Ser367 and Ser1893, which depends on DSBs sensor MRN, is required for complete activation of ATM [2, 3]. MRN allows ATM to be attached to the damaged sites of DNA strands. In turn, ATM monomers phosphorylate two components of the complex: Nbs1 and Mre11 [4]. Signal about DNA damage is transferred through ATM to other substrates, among them cell cycle checkpoint kinase Chk2 phosphorylated by ATM on Tyr68 [5, 6]. This phosphorylation leads to auto-phosphorylation of Chk2 on Tyr383 and Tyr387 and amplification of the signal about cell cycle arrest to conduct DNA repair [5, 7]. This phosphorylation leads to auto-phosphorylation of Chk2 on Tyr383 and Tyr387 and amplification of the signal about cell cycle arrest to conduct DNA repair [5, 7].

ATM triggers the activation of Mdm2 (E3 ubiquitin-protein ligase, double minute 2 homolog) – a natural inhibitor of p53 – probably through indirect phosphorylation of the protein kinase B (Akt) [8, 9, 10]. On the other hand, ATM acts as an activator of p53 not only directly through phosphorylation of this tumour suppressor on Ser15, but also indirectly through phosphorylation of Chk2 on Ser20 [11, 12]. Moreover, ATM influences nuclear mono-phosphorylated active form of Mdm2 by phosphorylation on Ser395, leading to inactivation of Mdm2 [13]. In turn, p53 indirectly up-regulates ATM transcription and directly inhibits Chk2 synthesis [14, 15, 16]. Chk2 plays an important role in regulation of p53-Mdm2 feedback loop by promoting degradation of Mdm2 and MdmX (Mdm family member; reviewed in [17]).

An important component of ATM/p53 signalling pathway is played by Wip1. Its main role is to inactivate phosphorylated forms of p53 (Ser15), ATM (Ser1981) and Chk2 (Tyr68), reviewed in [18, 19, 20]. Wip1 inactivates p53 also indirectly through dephosphorylation of multi-phosphorylated inactive form of Mdm2 (Ser395 phosphorylated earlier by ATM) [21, 22]. This special phosphatase is linked to ATM kinase also in another way: fully active ATM phosphorylates KSRP – KH-type splicing regulatory protein – on Ser670 and Ser132 [23, 24]. KSRP positively regulates miRNAs maturation, among them miR-16 responsible for down-regulation of Wip1 mRNA [25, 23, 26]. Another factor that links ATM with Wip1 is called CREB – cAMP response element-binding protein. When phosphorylated by active ATM (Tyr100, Ser111 and Ser121), CREB positively regulates synthesis of both ATM and Wip1 [27, 28, 29, 30, 31]. Furthermore, Wip1 transcription is up-regulated by p53 [32, 30]. Wip1 is also up-regulated by another transcription factor: NF-κB

(reviewed in [33, 19]). The main role of Wip1 in NF-κB pathway is inhibition of transcription of NF-κB-dependent genes, among them the genes encoding A20, IκBα, p53 and Wip1 proteins. In contrast, ATM activates NF-κB pathway via the IκB kinase complex [34, 35].

References

1. Bakkenist CJ, Kastan MB. DNA damage activates ATM through intermolecular autophosphorylation and dimer dissociation. Nature. 2003;421:499–506.
2. Kozlov SV, Graham ME, Peng C, Chen P, Robinson PJ, Lavin MF. Involvement of novel autophosphorylation sites in ATM activation. EMBO J. 2006;25:3504–3514.
3. Lee JH, Paull TT. ATM activation by DNA double-strand breaks through the Mre11-Rad50-Nbs1 complex. Science. 2005;308:551–554.
4. Uziel T, Lerenthal Y, Moyal L, Andegeko Y, Mittelman L, Shiloh Y. Requirement of the MRN complex for ATM activation by DNA damage. EMBO J. 2003;22:5612–5621.
5. Ahn JY, Schwarz JK, Piwnica-Worms H, Canman CE. Threonine 68 phosphorylation by ataxia telangiectasia mutated is required for efficient activation of Chk2 in response to ionizing radiation. Cancer Res. 2000;60:5934–5936.
6. Ward IM, Wu X, Chen J. Threonine 68 of Chk2 is phosphorylated at sites of DNA strand breaks. J Biol Chem. 2001;276:47755–47758.
7. Matsuoka S, Rotman G, Ogawa A, Shiloh Y, Tamai K, Elledge S J . Ataxia telangiectasia-mutated phosphorylates Chk2 in vivo and in vitro. Proc Natl Acad Sci. 2000;97:10389–10394.
8. Khosravi R, Maya R, Gottlieb T, Oren M, Shiloh Y, Shkedy D. Rapid ATM-dependent phosphorylation of MDM2 precedes p53 accumulation in response to DNA damage. Proc Natl Acad Sci. 1999;96:14973–14977.
9. Kubbutat M, Jones SN, Vousden KH. Regulation of p53 stability by Mdm2. Nature. 1997;387:299–303.
10. Xu N, Zhang Y, Gillespie DA. Akt: a double-edged sword in cell proliferation and genome stability. J Oncol. 2012;p. 9517–24.
11. Banin S, Moyal L, Shieh S, Taya Y, Anderson CW, Chessa L, et al. Enhanced phosphorylation of p53 by ATM in response to DNA damage. Science. 1998;281:1674–1677.
12. Chehab NH, Malikzay A, Stavridi ES, Halazonetis TD. Phosphorylation of Ser-20 mediates stabilization of human p53 in response to DNA damage. Proc Natl Acad Sci. 1999;96:13777–13782.
13. Maya R, Balass M, Kim ST, Shkedy D, Leal JFM, Shifman O, et al. ATM-dependent phosphorylation of Mdm2 on serine 395: role in p53 activation by DNA damage. Genes Dev. 2001;15:1067–1077.
14. Craig AL, Holcakova J, Finlan LE, Nekulova M, Hrstka R, Gueven N, et al. Delta Np63 transcriptionally regulates ATM to control p53 Serine-15 phosphorylation. Mol Cancer. 2010;9(195).
15. Huang Y, Guerrero-Preston R, Ratovitski EA. Phospho-delta Np63 alfa-dependent regulation of autophagic signaling through transcription and micro-RNA modulation. Cell Cycle. 2012;11:1247–1259.
16. Matsui T, Katsuno Y, Inoue T, Fujita F, Joh T, Niida H, et al. Negative regulation of Chk2 expression by p53 is dependent on the CCAAT-binding transcription factor NF-Y. J Biol Chem. 2004;279:25093–25100.
17. Ahn JY, Urist M, Prives C. The Chk2 protein kinase. DNA Repair. 2004;3:1039–1047.
18. Lu X, Ma O, Nguyen TA, Jones SN, Oren M, Donehower LA. The Wip1 Phosphatase acts as a gatekeeper in the p53-Mdm2 autoregulatory loop. Cancer cell. 2007;12:342–354.
19. Lowe J, Cha H, Lee MO, Mazur SJ, Appella E, Fornace AJ Jr. Regulation of the Wip1 phosphatase and its effects on the stress response. Front Biosci. 2012;17:1480–1498.
20. Shimada M, Nakanishi M. Response to DNA damage: why do we need to focus on protein phosphatases? Front Oncol. 2013;3(8).
21. Lu X, Nguyen TA, Zhang X, Donehower LA. The Wip1 phosphatase and Mdm2: cracking the "Wip" on p53 stability. Cell Cycle. 2008;7:164–168.
22. Zhang X, Lin L, Guo H, Yang J, Jones SN, Jochemsen A, et al. Phosphorylation and degradation of MdmX is inhibited by Wip1 phosphatase in the DNA damage response. Cancer Res. 2009;69:7960–7968.
23. Zhang X, Wan G, Berger FG, He X, Lu X. The ATM kinase induces microRNA biogenesis in the DNA damage response. Mol Cell. 2011;41:371–383.
24. Liu Y, Liu Q. ATM signals miRNA biogenesis through KSRP. Mol Cell. 2011;41:367–368.
25. Trabucchi M, Briata P, Garcia-Mayoral M, Haase AD, Filipowicz W, Ramos A, et al. The RNA-binding protein KSRP promotes the biogenesis of a subset of microRNAs. Nature. 2009;459:1010–1014.
26. Zhang X, Wan G, Mlotshwa S, Vance V, Berger FG, Chen H, et al. Oncogenic Wip1 phosphatase is inhibited by miR-16 in the DNA damage signaling pathway. Cancer Res. 2010;70:7176–7186.
27. Shi Y, Venkataraman SL, Dodson GE, Mabb AM, LeBlanc S, Tibbetts RS. Direct regulation of CREB transcriptional activity by ATM in response to genotoxic stress. Proc Natl Acad Sci U S A. 2004;101:5898–5903.
28. Shaywitz AJ, Greenberg ME. CREB: a stimulus-induced transcription factor activated by a diverse array of extracellular signals. Annu Rev Biochem. 1999;68:821–861.
29. Mayr B, Montminy M. Transcriptional regulation by the phosphorylation-dependent factor CREB. Nat Rev Mol Cell Biol. 2001;2:599–609.
30. Rossi M, Demidov ON, Anderson CW, Appella E, Mazur SJ. Induction of PPM1D following DNA-damaging treatments through a conserved p53 response element coincides with a shift in the use of transcription initiation sites. Nucleic Acids Res. 2008;36:7168–7180.
31. Zhang X, Odom DT, Koo SH, Conkright MD, Canettieri G, Best J, et al. Genome-wide analysis of cAMP-response element binding protein occupancy, phosphorylation & target gene activation in human tissues. Proc Natl Acad Sci U S A. 2005;102:4459–4464.

32. Fiscella M, Zhang H, Fan S, Sakaguchi K, Shen S, Mercer WE, et al. Wip1, a novel human protein phosphatase that is induced in response to ionizing radiation in a p53-dependent manner. Proc Natl Acad Sci. 1997;94:6048–6053.
33. Salminen A, Kaarniranta K. Control of p53 and NF-kB signaling by WIP1 and MIF: role in cellular senescence and organismal aging. Cell Signal. 2011;23:747–752.
34. Li N, Banin S, Ouyang H, Li GC, Courtois G, Shiloh Y, et al. ATM is required for IkB kinase (IKK) activation in response to DNA double strand breaks. J Biol Chem. 2001;276:8898–8903.
35. Huang TT, Wuerzberger-Davis SM, Wu ZH, Miyamoto S. Sequential modification of NEMO/IKK by SUMO-1 and ubiquitin mediates NF-kB activation by genotoxic stress. Cell. 2003;115:565–576.
