# Supplementary material for: A novel mathematical model of ATM/p53/NF- κB pathways points to the importance of the DDR switch-off mechanisms
Source: BMC Syst Biol. 2016 Aug 15;10:75. doi: 10.1186/s12918-016-0293-0 (PMC4986247; doi:10.1186/s12918-016-0293-0)
Supplement: Additional file 6 — Cell fate decision. Description of the procedure of determining the proper thresholds for the decision about the cell fate in the model. (PDF 103 kb) [file 12918_2016_293_MOESM6_ESM.pdf]

A novel mathematical model of ATM/p53/NF-κB pathways points to the importance of the DDR switch-off mechanisms

ADDITIONAL FILE
Cell fate decision - determining the thresholds

To find the proper thresholds, first we fitted our model to the results of flow cytometry experiments that provided the number of apoptotic cells. We determined the best fit for active p53 level equal to 72000, Bax to 32000 and p21 to 27000 molecules per cell. We assumed that if the cell did not die during the time t after irradiation, it could initiate the formation of the colony, what indicates that this cell can divide at least once before its death. Marahaini *et al.* [1] reported that the time of the cell cycle from G1 phase to division of U2-OS cells is equal 32±3 hours, therefore the time t should be lower than this value. We found that the best results are obtained for $t = 28$ hours. In our experiments, as viable we considered only these cells which form the colonies of at least 30 cells. Assuming that each cell in the colony divides with the same rate, 30 cells threshold is equal to minimum 5 divisions, what gives the time span of 145-175 hours. Colonies were counted after 10 days of incubation what is equal to 240 hours after treatment with IR. The above numbers indicate that if the cell does not die before the first division and if the mitotic cycle of that cell is continuously blocked for 65-95 hours after irradiation during 240 hours of simulation, it would not have a time to form colonies large enough. In our *in silico* experiments, we received the best viability fit for the length of cell cycle blockade equal 63.9 hours. These results are reasonable for cells assuming that the cycle requires some time to restart.

References

1. Marahaini M, Thirumulu PK, Shyamoli M. Cell Proliferation Study of Human Osteosarcoma Cell Line (U2OS) using Alamar Blue Assayand Live Cell Imaging. IOSR-JDMS. 2013;8:60–65.
