# Supplementary figures and images for: A novel mathematical model of ATM/p53/NF- κB pathways points to the importance of the DDR switch-off mechanisms
Source: BMC Syst Biol. 2016 Aug 15;10:75. doi: 10.1186/s12918-016-0293-0 (PMC4986247; doi:10.1186/s12918-016-0293-0)

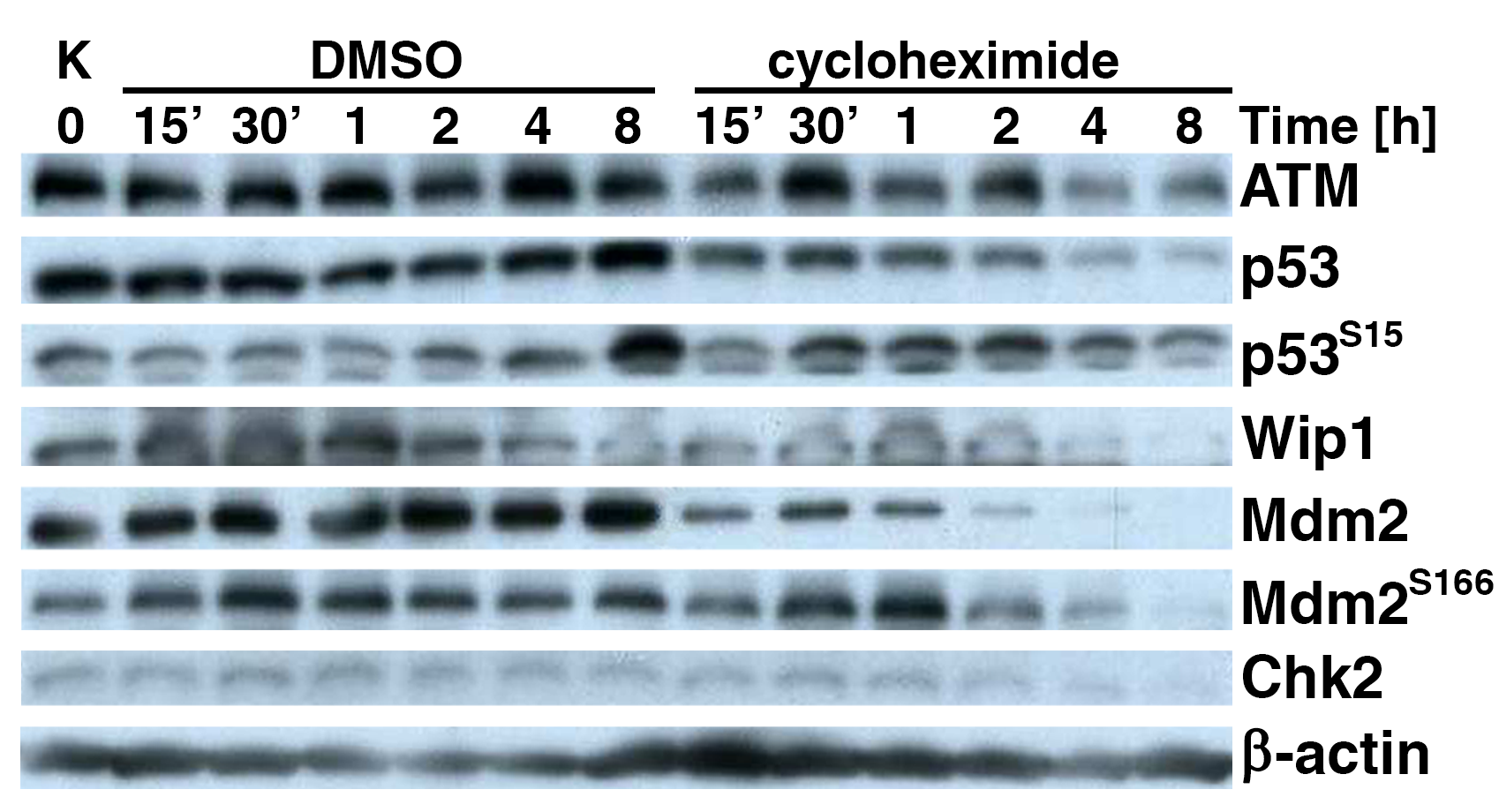

Supplement: Additional file 7 — Determining the half-life time of proteins included in the model. Immunoblots of total ATM, unphosphorylated p53, p53 phosphorylated on Ser15, Wip1, Hdm2, Hdm2 phosphorylated on Ser166, Chk2. (PNG 738 kb) [file 12918_2016_293_MOESM7_ESM.png]

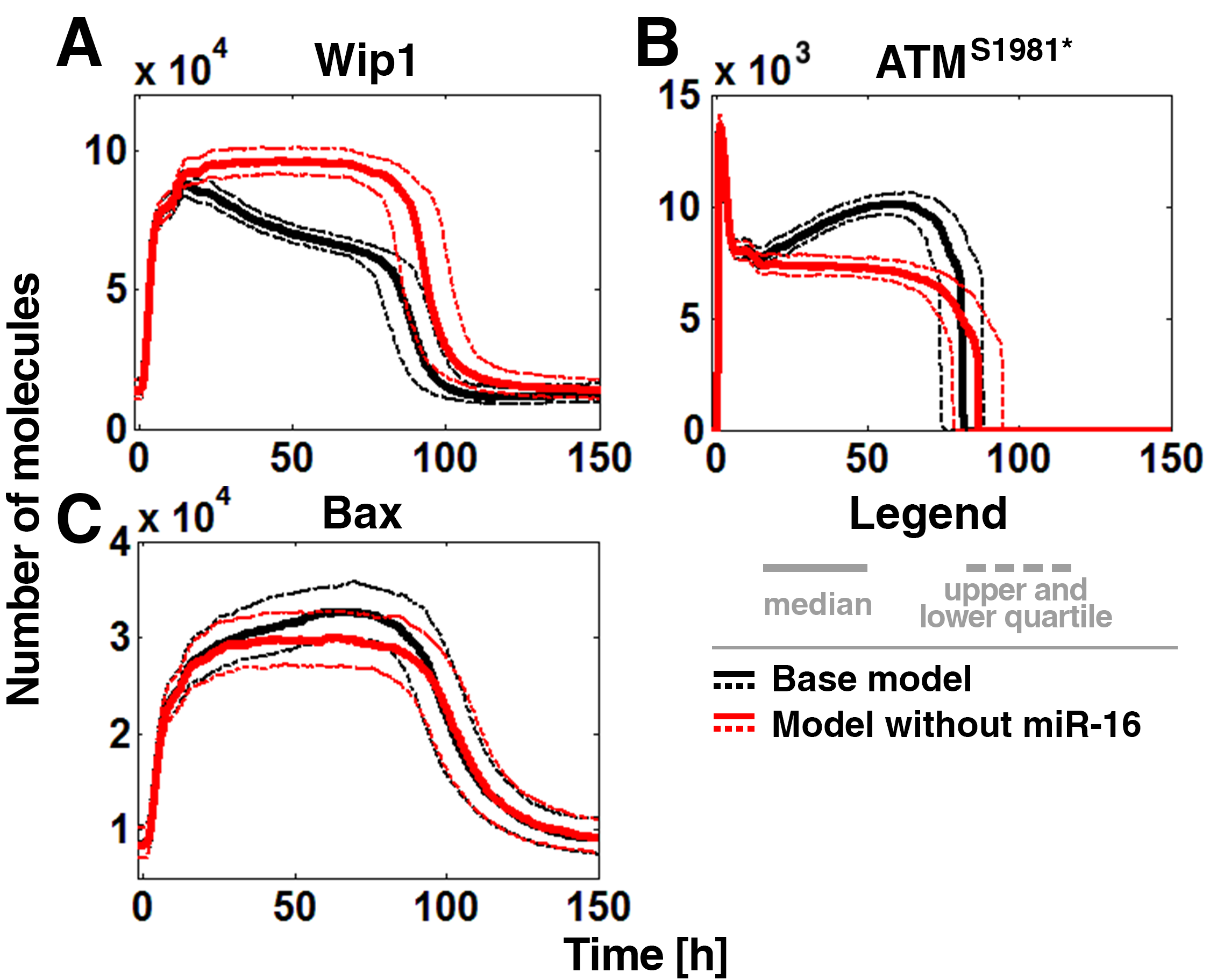

Supplement: Additional file 9 — Impact of miR-16. Impact of miR-16 deletion on irradiated cells. (PNG 2089 kb) [file 12918_2016_293_MOESM9_ESM.png]
