# Supplementary material for: A novel mathematical model of ATM/p53/NF- κB pathways points to the importance of the DDR switch-off mechanisms
Source: BMC Syst Biol. 2016 Aug 15;10:75. doi: 10.1186/s12918-016-0293-0 (PMC4986247; doi:10.1186/s12918-016-0293-0)
Supplement: Additional file 8 — Sensitivity analysis. Description and results of the sensitivity analysis of the fitted parameters. (PDF 1413 kb) [file 12918_2016_293_MOESM8_ESM.pdf]

A novel mathematical model of ATM/p53/NF-κB pathways points to the importance of the DDR switch-off mechanisms

ADDITIONAL FILE
Sensitivity analysis of the fitted parameters

We performed local sensitivity analysis of the fitted parameters according to the procedure described in [1]. Let the analysed model be described by the Eq. (1):

$$\frac{d\mathbf{X}}{dt} = \mathbf{f}(\mathbf{X}, \mathbf{u}, \mathbf{p}) \tag{1}$$

with a solution described as:

$$\mathbf{X}(\mathbf{p_n}, \mathrm{t}) \tag{2}$$

where: $\mathbf{X}$=$[x_1 x_2 \cdots x_n]^T$ - state vector with x_i
x_i - number of molecules of type i
$\mathbf{u}$ - input variable
$\mathbf{p}$ - model parameters
p_n - nominal parameter vector

The sensitivity function s_{ij} changing over time describes the influence of each i-th parameter on each j-th variable:

$$s_{ij} = \frac{\partial x_i}{\partial p_j} \tag{3}$$

The absolute sensitivity matrix is given by:

$$\mathbf{S} = \frac{\partial \mathbf{X}}{\partial \mathbf{p}} = \begin{bmatrix} s_{11} & s_{12} & \cdots & s_{1m} \\ s_{21} & s_{22} & \cdots & s_{2m} \\ \vdots & \vdots & \ddots & \vdots \\ s_{n1} & s_{n2} & \cdots & s_{nm} \end{bmatrix} \tag{4}$$

Analytical solution of Eq. (2) is is very time-consuming and difficult to obtain, therefore the sensivity coefficients were calculated using a direct differential method:

$$\frac{d}{dt}\frac{\partial \mathbf{X}}{\partial p_j} = \frac{\partial f}{\partial \mathbf{X}}\frac{\partial \mathbf{X}}{\partial p_j} + \frac{\partial f}{\partial p_j} = J \cdot S_j + F_j \tag{5}$$

where J states for Jacobi matrix given by:

$$J = \frac{\partial f}{\partial \mathbf{X}} = \begin{bmatrix} \frac{\partial f_1}{\partial x_1} & \frac{\partial f_1}{\partial x_2} & \cdots & \frac{\partial f_1}{\partial x_n} \\ \frac{\partial f_2}{\partial x_1} & \frac{\partial f_2}{\partial x_2} & \cdots & \frac{\partial f_2}{\partial x_n} \\ \vdots & \vdots & \ddots & \vdots \\ \frac{\partial f_n}{\partial x_1} & \frac{\partial f_n}{\partial x_2} & \cdots & \frac{\partial f_n}{\partial x_n} \end{bmatrix} \tag{6}$$

F_j is the parametric Jacobi matrix for j-th parameter:

$$F_j = \frac{\partial f}{\partial p_j} = \begin{bmatrix} \frac{\partial f_1}{\partial p_j} \\ \frac{\partial f_2}{\partial p_j} \\ \vdots \\ \frac{\partial f_n}{\partial p_j} \end{bmatrix} \tag{7}$$

and S_j is the sensivity vector for j-th parameter:

$$S_j = \frac{\partial \mathbf{X}}{\partial p_j} = \begin{bmatrix} s_{1j} \\ s_{2j} \\ \vdots \\ s_{nj} \end{bmatrix} \tag{8}$$

Equations (1) and (5) can be combined in order to calculate sensitivity coefficients:

$$\begin{aligned} \dot{\mathbf{X}} &= f(\mathbf{X},\mathbf{p}, u, t) \\ \dot{S_j} &= J \cdot S_j + F_j \end{aligned} \tag{9}$$

The initial conditions of sensitivity coefficients are given by:

$$S_j(0) = \frac{\partial x(0)}{\partial p_j} \tag{10}$$

To investigate the response of the system with an initial state as an equilibrium, the initial conditions should depend on the model parameters. To find the initial values for sensitivity coefficients, we simulated our original model (with known initial values) together with sensitivity functions (with initial values equal zero). Simulations end when the equilibrium state was reached. The values obtained by the described method became the initial conditions for the sensitivity functions.

In order to find the time courses for the sensitivity functions we simulated them together with original equations from our model. The results were normalized (11) to find the relative influence of the parameters on the system behaviour regardless to the scale of the parameters and the variables.

$$\overline{s_{ij}} = \frac{\partial x_i}{\partial p_j} \cdot \frac{p_j}{x_i} \tag{11}$$

We measured the influence of the j-th parameter for the i-th state variable:

$$S_{ij}* = \frac{1}{N}\sqrt{\sum_{k=1}^{N} |S_{ij}(k)|^2} \qquad (12)$$

where N is the number of simulation steps.

The calculated values allow us to obtain a ranking of the parameters from the most robust to the parameters for which the system is the most sensitive.

As the reference for our analysis, we chose the proteins with the levels known to be crucial for cell fate determination: phosphorylated p53 (p53p), p21 and Bax (Figs. 1-3). 73 parameters were fitted in our model. Although this number seems to be high, most of the parameters from our model can be fitted in separate modules to the real biological data, such as fold change of the protein level after IR or size of apoptotic fraction after various doses of IR, what makes this fit very reliable.

In this supplement we show how the fitted parameters influence the system response. The *sensitivity* value on the plots or *value* in the table indicate how much the individual protein level changes in response to a variation of the value of individual parameter. For example, the influence of $wq1$ parameter on p53p level is equal 0.1084, what means that when the value of $wq1$ changes around 20%, p53p level will change around $(0.2 \cdot 0.1084) \cdot 100\% = 2.168\%$. As one can notice, there is only 16 parameters of total 73 that significantly influence the level of the marker proteins (value > 0.1 in the tables 1 and 2). Changes of six of these parameters have higher effect on the system response, as the indicator of this fact is higher than 0.5. These parameters are $ps2$, $pd9$, $bs1$, $bs2$, $bt1$ and $bt2$, and will be discussed in more details further in this Supplement.

$bs1$ and $bt1$ are transcription and translation coefficients for Bax, while $bs2$ and $bt2$ for p21. These parameters are part of the separate module that does not influence any part of the main model, but only the final cell fate decision. Therefore, the parameters are important for the levels of Bax and p21, but not for p53p (Tab. 1). Due to the fact that in our model p21 and Bax levels are responsible for cell cycle blockade/apoptotic decision, and this decision depends on the given thresholds cross, the transcription and translation parameters should be considered in conjunction with appropriate thresholds. Every change in the values of these parameters should be followed with a proper change in the threshold value. Because Bax and p21 levels follow simple two-step-production dynamics without any feedback, given fit, even if received from only 24 points (12 for Bax and 12 for p21), would be accurate.

The cell fate decision in our model depends on the levels of p53p, Bax and p21. Therefore, because p21 and Bax are transcriptionally dependent on p53p, their levels are crucial for the model output. The main influence on p53p level is given by its inhibitor Mdm2. Thus, it is not surprising that parameters responsible for Mdm2 level are on the top places in the parameters ranking for all decision proteins. Among them the main influence is given by the rate of Mdm2 mRNA synthesis ($ps2$) and the rate of Mdm2 degradation by Chk2 ($pd9$). Please notice that we have

experimentally determined the parameters of degradation for IR untreated cells and we know the exact levels of Mdm2 and p53 in U2-OS cells [2]. Once again, this fact makes the parameters of production for Mdm2 easier to fit, because other parameters involved in p53 and Mdm2 levels determination in cases without any stimuli are known and make this fit reliable. Concerning $pd9$ parameter, we have experimental measurements how the Mdm2, Chk2 and p53 levels change after the irradiation. Knowing the parameters value for the steady state without IR, we can reliably fit the remaining parameters to properly describe not only change of the levels of the variables, but also dynamics of this change. However, we do not claim the uniqueness of our fitting, but we show that the fitting procedure is not "all-at-once" case. It is rather a "piece-by-piece" method, where a complex model is decomposed into smaller modules easier to fit to experimental data.

Here, we described how the normalized sensitivity functions value changes over time for the parameters from the top, middle and bottom of our parameters ranking (Fig. 4). As expected, the impact of the parameters from the top of the ranking on the protein level is greater than the impact of the parameters with lower place in the ranking. The higher influence of the parameters value on the protein levels is shortly after irradiation $(0 < t < 10h)$, when after that time it decreases strongly. This observation is important for development of our model, because, as shown in Fig. 5, at this time the Bax level is far below the threshold, thus the influence of the parameters on the apoptotic decision is limited. Similarly, p21 level is much above the threshold, so the influence of the parameters on the cell cycle decision is also limited. Although p53p level seems to stay below the threshold after the initial peak, in fact every single cell exhibits strong p53p oscillations at time $t > 10h$. These oscillations are not visible on the median level, due to the strong desynchronization between the cells. However, the influence of the parameters on the model output is still noticeable. Nevertheless, due to the fact that the decision about the cell fate is taken at the time when the sensitivity of the parameters on the levels of the decision proteins is close to zero, that influence is limited.

References

1. Puszynski K, Lachor P, Kardynska M, Smieja J. Sensitivity analysis of deterministic signaling pathways models. Bull Pol Ac: Tech. 2012;60(3):471–479.
2. Wang YV, Wade M, Wong E, Li YC, Rodewald LW, Wahl GM. Quantitative analyses reveal the importance of regulated Hdmx degradation for p53 activation. Proc Natl Acad Sci U S A. 2007;104:12365–12370.

Table 1 Parameters ranking according to the median sensitivity value, part 1.

	p53p		Bax		p21	
no.	name	value	name	value	name	value
1	ps2	-0.8781	bt1	1	bt2	1
2	pd9	0.5957	bs1	0.9999	bs2	1
3	ps1	0.3246	ps2	-0.7133	ps2	-0.7584
4	pt1	0.3246	pd9	0.5428	pd9	0.6142
5	pq3	-0.3229	pt1	0.2328	ps1	0.2547
6	pd3	-0.2732	ps1	0.2328	pt1	0.2547
7	pd5	-0.2685	wt1	-0.2250	wt1	-0.2487
8	wt1	-0.2094	ws1	-0.2250	ws1	-0.2487
9	ws1	-0.2094	pd3	-0.2041	pd3	-0.2261
10	pa1	0.1855	pd5	-0.1537	pd5	-0.1628
11	pi1	-0.1828	wq1	0.1277	wq1	0.1420
12	pq5	-0.1605	pa6	0.1255	pa6	0.1346
13	pq1	0.1566	pa5	-0.1190	pa5	-0.1283
14	pc4	0.1473	pa1	0.1184	pq3	0.1275
15	pa4	-0.1187	pq3	0.1165	pa1	0.1260
16	pa6	0.1176	pq5	-0.1132	pq5	-0.1242
17	pa5	-0.1145	pi1	-0.1104	pi1	-0.1189
18	wq1	0.1084	pq1	0.1077	pq1	0.1184
19	pa8	-0.1066	mt1	-0.0770	mt1	0.0956
20	mt1	0.1058	ms1	0.0770	ms1	0.0956
21	ms1	0.1058	pa4	-0.0743	mq2	-0.0862
22	pc3	0.1003	mq2	0.0722	pa4	-0.0815
23	pa9	-0.0732	pc1	-0.0691	pc1	-0.0767
24	pa2	0.0721	pa8	-0.0675	pc4	0.0745
25	pa3	0.0716	pc4	0.0644	pa8	-0.0740
26	pc1	-0.0655	pc3	-0.0602	pc3	0.0675
27	mq2	-0.0634	pa3	-0.0544	wd3	0.0645
28	mc2	-0.0627	pa2	0.0544	wa2	0.0645
29	mq3	-0.0507	pa9	0.0534	ws2	0.0645
30	mq1	0.0486	wd3	0.0477	pa2	0.0618
31	wd3	0.0424	wa2	0.0477	pa3	0.0618
32	wa2	0.0424	ws2	0.0477	pa9	-0.0607
33	ws2	0.0424	mc2	0.0411	wd6	-0.0567
34	wd6	-0.0377	wd6	-0.0404	mc2	-0.0552
35	ma3	0.0360	ma5	0.0382	wi1	0.0497
36	mc5	0.0346	mc5	-0.0375	wd5	-0.0459
37	ma5	-0.0331	wi1	0.0367	ma5	-0.0449

Table 2 Parameters ranking according to the median sensitivity value, part 2.

	p53p		Bax		p21	
no.	name	value	name	value	name	value
38	wi1	0.0327	mq3	-0.0335	mc5	0.0448
39	wd5	-0.0310	wa1	0.0326	we1	-0.0435
40	we1	-0.0289	wd5	-0.0314	wa1	0.0428
41	wa1	0.0278	we1	0.0309	mq3	-0.0403
42	ma4	0.0264	mq1	0.0285	wc1	-0.0334
43	wc1	-0.0221	wc1	-0.0241	ma3	0.0329
44	mc4	-0.0182	ma3	0.0235	mq1	0.0328
45	ma6	0.0165	ma4	-0.0191	ma4	0.0254
46	nq1	-0.0103	mc4	-0.0128	mc4	-0.0169
47	nk2	0.0094	ma6	-0.0121	ma6	0.0161
48	nq2	-0.0080	nq1	0.0041	nq1	-0.0045
49	nt1	0.0070	nk2	-0.0035	nk2	0.0039
50	na6	-0.0065	nq2	0.0031	nq2	-0.0035
51	nt2	0.0037	nt1	-0.0022	nt1	0.0026
52	nm4	-0.0037	na6	0.0012	na6	-0.0017
53	nd1	0.0019	nt2	0.0007	nt2	0.0010
54	ni2	0.0015	nm4	0.0007	nm4	-0.0010
55	pm3	0.0014	ma7	0.0007	ma7	0.0009
56	nm1	0.0012	nd1	-0.0005	nd1	-0.0007
57	pm2	0.0012	nm1	0.0005	nm1	0.0006
58	mt2	0.0012	ni2	-0.0003	ni2	0.0004
59	ms2	0.0012	pm3	0.0003	pm3	0.0004
60	ma7	0.0010	ma1	-0.0001	ma1	0.0003
61	ma1	0.0009	pm2	0.0001	mm2	-0.0002
62	mm2	-0.0008	mt2	0.0001	pm2	0.0002
63	pm1	0.0006	ms2	0.0001	mt2	0.0002
64	mm3	0.0001	mm2	-0.0001	ms2	0.0002
65	mc1	0	mc1	0	mc1	-0.0001
66	mc3	0	mc3	0	pm1	0
67	mm1	0	pm1	0	mc3	0
68	nm2	0	mm3	0	mm3	0
69	wd2	0	mm1	0	mm1	0
70	bs1	0	nm2	0	nm2	0
71	bt1	0	wd2	0	wd2	0
72	bs2	0	bs2	0	bs1	0
73	bt2	0	bt2	0	bt1	0

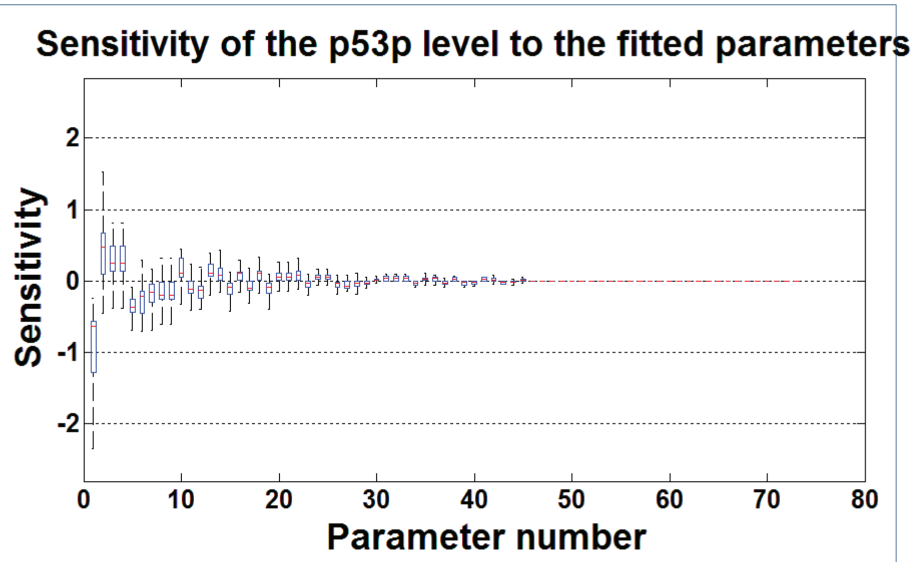

Figure 1 Sensitivity of the phosphorylated p53 level to the fitted parameters. Classic boxplots with median, quartiles and max-min values.

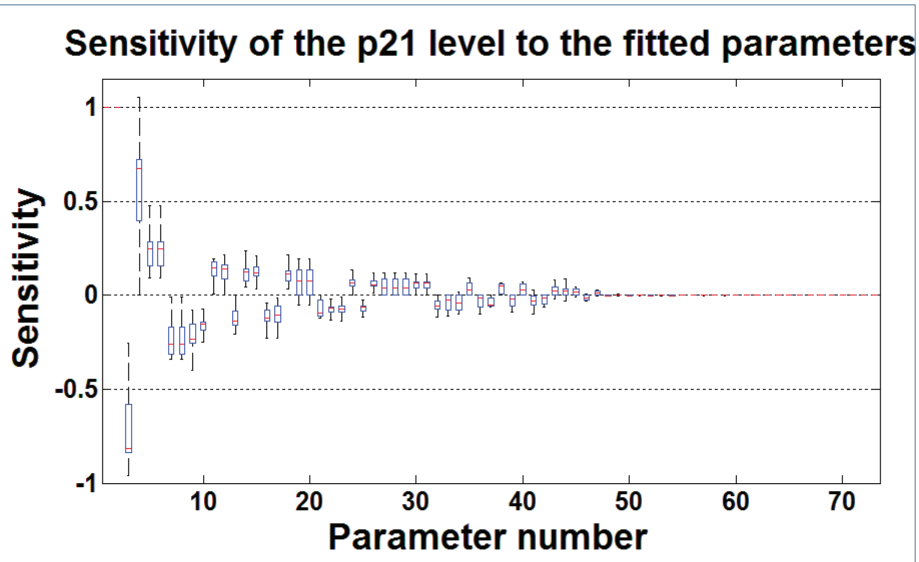

Figure 2 Sensitivity of the p21 level to the fitted parameters. Classic boxplots with median, quartiles and max-min values.

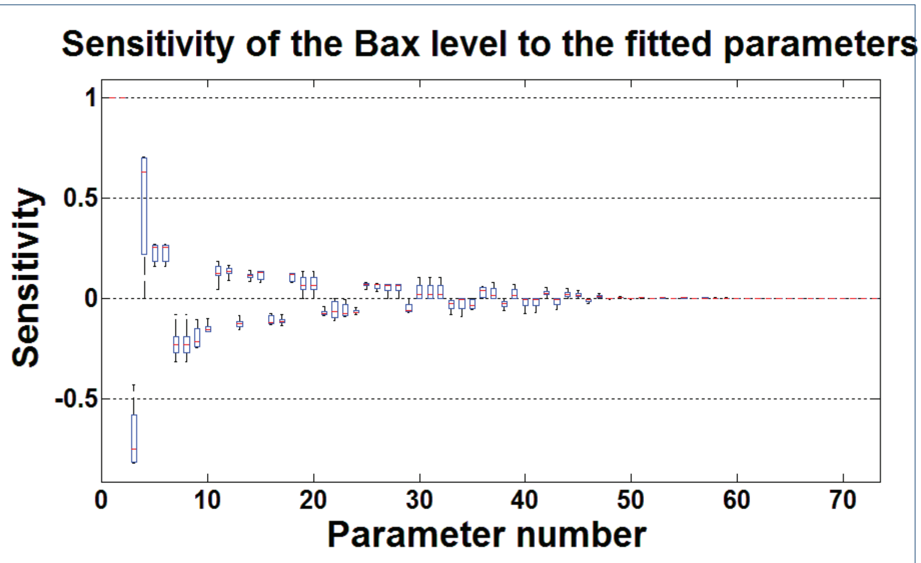

Figure 3 Sensitivity of the Bax level to the fitted parameters. Classic boxplots with median, quartiles and max-min values.

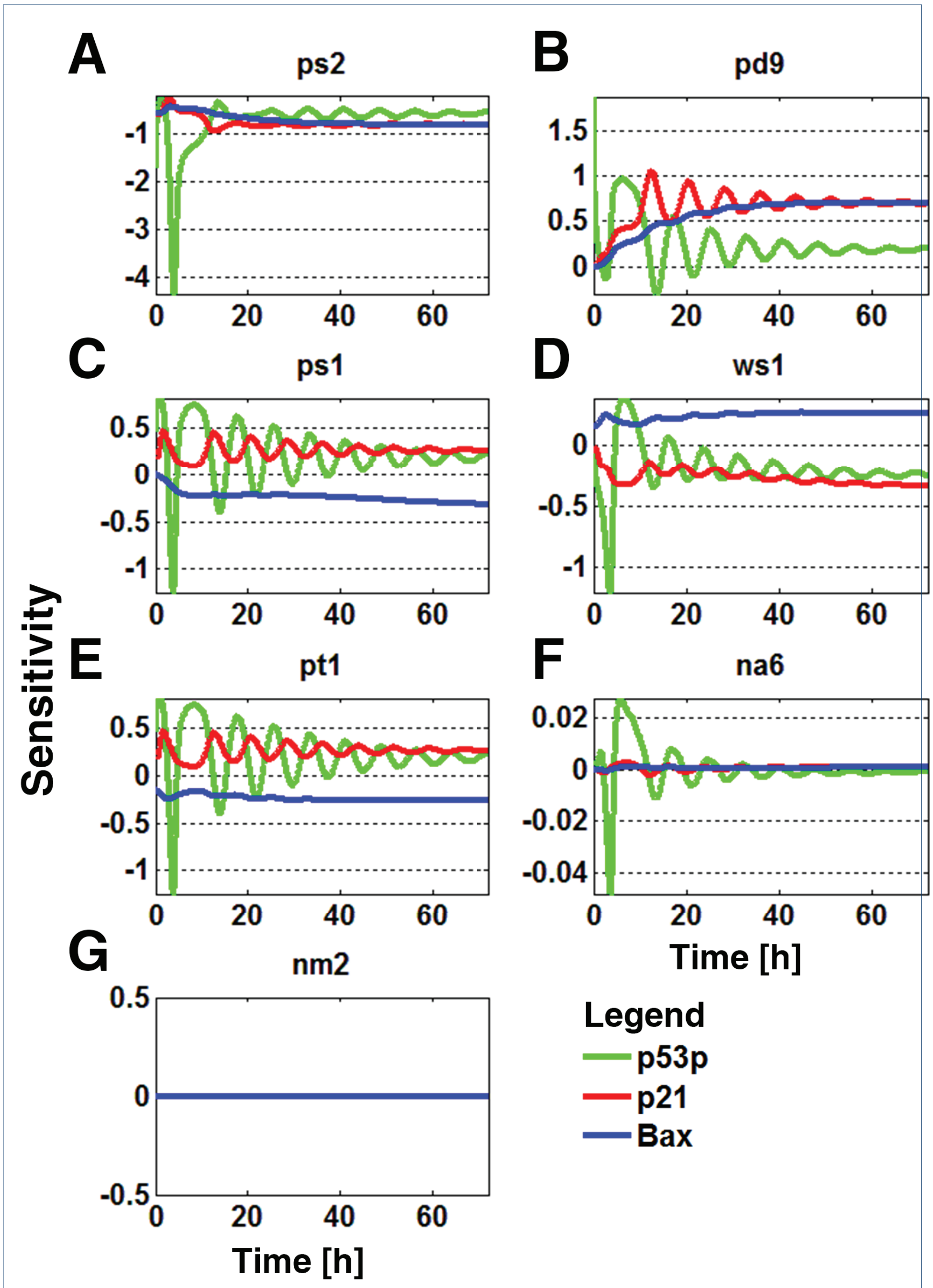

Figure 4 Normalized sensitivity functions for the parameters from different position in the ranking. Parameters meaning: ps2-synthesis of Mdm2 mRNA, pd9-Mdm2 degradation by Chk2, ps1-synthesis of p53 mRNA, ws1-WIP1 synthesis rate, pt1-p53 translation rate, na6-activation of IKK by ATM, nm2-coefficient for IKKK inhibition by A20

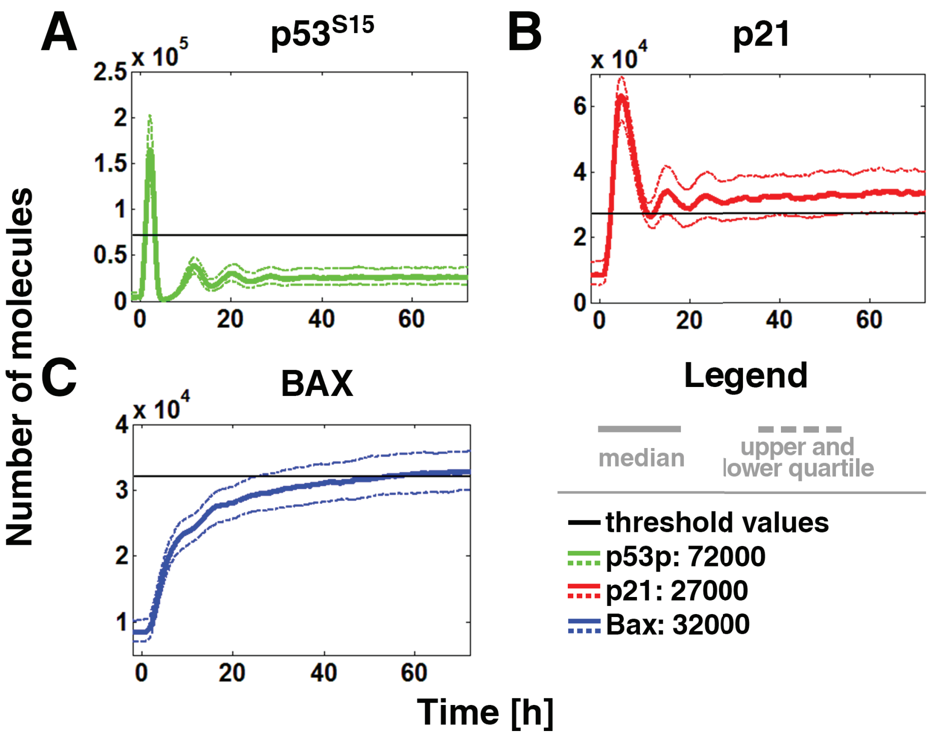

Figure 5 Median and quartiles of the p53p, p21 and Bax level in response to 10 Gy.
