# Supplementary material for: A novel mathematical model of ATM/p53/NF- κB pathways points to the importance of the DDR switch-off mechanisms
Source: BMC Syst Biol. 2016 Aug 15;10:75. doi: 10.1186/s12918-016-0293-0 (PMC4986247; doi:10.1186/s12918-016-0293-0)
Supplement: Additional file 10 — Methods. Detailed description about the experimental methods described in the article. (PDF 132 kb) [file 12918_2016_293_MOESM10_ESM.pdf]

# A novel mathematical model of ATM/p53/NF-$\kappa$B pathways points to the importance of the DDR switch-off mechanisms

# ADDITIONAL FILE
# Methods

**mRNA half-life analysis.** Degradation rates of the transcripts were determined using qPCR method. Cells, were harvested and total RNA was isolated from cells using Total RNA Isolation kit (A&A Biotechnology) and purified by DNase I digestion. Fifty ng of RNA was used for cDNA synthesis with random hexamers (Thermo), and then for gene-specific QRT-PCR analysis. Real-time PCR reactions were performed using Real Time PCR Master Mix SYBR (A&A Biotechnology) and CFX-96TM system (BioRad). The primer pairs were used to analyse transcripts of ATM: 5'-catgggcattacgggtgttg-3', 5'-catcttccggcctctgctgt-3'; CHEK2: 5'-acccgtggcttcaggatga-3', 5'-acacagctgggcgctttgtg-3'; WIP1 (PPM1D): 5'-ctcaatgtgccaggaccaagag-3', 5'-tatctgctcggagcatacgctg-3'; TP53: 5'-cctggattggcagccagact-3', 5'-tctgaaaatgtttcctgactcagagg-3'; HDM2: 5'-cgtgaaggaaactggggagt-3', 5'-ccgaagctggaatctgtgag-3'; GAPDH (reference gene): 5'-cgtcttcaccaccatggaga-3', 5'-ggccatcacgccacagttt-3'.

**Western blots.** In order to verify the results of the simulation analysis of the presented model, the data of change in level of proteins over time was determined by using western blot assay. Cells were washed with PBS, trypsynized and then suspended in RIPA lysis buffer (1x PBS, 1% NP-40, 0.5% SDC, 0.1% SDS) supplemented with protease inhibitors cocktail (Roche) and Phosphatase Inhibitor Cocktail 2 (Sigma-Aldrich). Cells were incubated in the lysis buffer for 20 minutes on ice and then the suspension was centrifuged (21 000 x g, 4°C, 20 min). Total protein amount in obtained whole-cell lysates was quantified using the Bio-Rad Protein Assay. Samples containing 10-40 µg of proteins were separated by SDS-PAGE (6% or 10%) and electrotransferred onto PVDF membranes. The membranes were blocked for 1 h at room temperature in 5% solution of skim milk in TBST 0.1%, and then incubated overnight at 4°C with the primary antibodies: for the kinetic analysis after treatments the following rabbit polyclonal antibodies were used: anti-p53Ser15, anti-WIP1, anti-CHK2Thr68, anti-HDM2Ser166, anti-$\beta$-actin (Cell Signaling Technology); for determining protein half-lives the following antibodies were used: anti-ATM, anti-CHK2, anti-WIP1, anti-p53Ser15, anti-HDM2Ser166 (Cell Signaling Technology), anti-p53 and anti-HDM2 (Santa Cruz). Following the triple TBST washing the membranes were incubated for 1 h at room temperature with HPR-conjugated secondary antibody, and then specific protein bands were detected using SuperSignal West Pico Chemiluminescent Substrate (Thermo Scientific).

**$\gamma$-H2AX detection.** Determination of the amount of DNA DSBs was based on the analysis of the $\gamma$-H2AX foci counting assay. Cells were fixed at specific time

points to study $\gamma$-H2AX. Cells were fixed in 4% formaldehyde (HCHO:PBS) for 30 minutes and in 3% $H_2O_2$ ($H_2O_2$:$CH_3OH$) for 10 minutes at room temperature. After fixation cells were permeabilized with 0.1% Triton X100 (Triton X100:0,01M citrate buffer) and then blocked with 3% BSA solution (BSA:PBS). Cells were then stained with anti-$\gamma$-H2AX antibody (Millipore) and biotinylated anti-mouse secondary antibody (Vector). Aggregations of $\gamma$-H2AX were visualized using streptavidin, conjugated with Texas Red (1:250 in 10mM Hepes). Coverslips were mounted with VECTASHIELD®Mounting Medium containing DAPI, to counterstain cellular nuclei. $\gamma$-H2AX foci were scored manually by the same operator throughout the cell nuclei using a Nikon Eclipse 80i fluorescence microscope with 100X objective and the average number of foci per cell was calculated from a minimum of 250 cells per time point. Experimental data represent the average of three independent experiments.

**Quantification of degradation rates.** For degradation rates of transcripts, the normalized threshold cycle values obtained from qPCR experiments were used. The regression analysis with logarithmic approximation was performed with the start point of the highest expression value, and the half-life time was quantified. Degradation rate was quantified as a quotient of the natural logarithm of two and the half-life time in seconds. For degradation rates of proteins, the amount of the proteins from western blot experiments was quantified using extended version of the application for analysis of the images from western blot assay [1], developed by the authors. The relative level of the proteins was counted by performing normalization of the material to untreated cells from time zero, and the degradation rate was quantified as described in case of degradation of the transcripts.

**Flow cytometry.** Flow cytometry was used to determinate the amount of apoptotic cells (Sub-G1 fraction) treated with several different doses of IR. Cells were collected after a brief incubation with trypsin solution (Sigma), centrifuged for 2 minutes at 600 x g, washed in PBS, fixed in 70% ethanol and stored at -20°C. Fixed cells were washed twice in PBS and re-suspended in PBS containing 50 µg/ml propidium iodide and 0.2 mg/ml RNase A for 15 minutes at room temperature, and then immediately analysed using a FACScan flow cytometer (Becton Dickinson). Ten thousands cells were counted in each sample for assessment of the cell-cycle phase distribution. The percentage of apoptotic cells was calculated from the sub-G1 peak of the DNA histograms using CellQuest software (Becton Dickinson). Relative frequencies of apoptotic cells were expressed as ratios of the percentage of sub-G1 cells in treated and in appropriate untreated control cultures.

**Clonogenic cell survival assay.** U2-OS cells were seeded in plates at the required density in DMEM medium (SIGMA), cultured for 48 hour and then irradiated with ionizing radiation at the following doses: 0, 2, 4, 6 and 8 Gy. Cells were allowed to grow in fresh McCoy's medium, until they formed colonies for 10 days. After this time survived colonies were fixed with methanol:aceton (1:1) for 2 minutes in RT, stained with crystal violet for 15 minutes in 37°C and washed with deionized water. For each sample, large colonies (>30 cells) were counted from three independent experiments and IR-treated samples were refereed to untreated control cells (0 Gy).

**References**

1. Jonak K, Jedrasiak K, Polanski A, Puszynski K. Application of image processing algorithms in proteomics: automatic analysis of 2-d gel electrophoresis images from western blot assay. Computer Vision and Graphics. 2012;p. 433–440.
